# The novel genus, ‘*Candidatus* Phosphoribacter’, previously identified as *Tetrasphaera*, is the dominant polyphosphate accumulating lineage in EBPR wastewater treatment plants worldwide

**DOI:** 10.1038/s41396-022-01212-z

**Published:** 2022-02-25

**Authors:** C. M. Singleton, F. Petriglieri, K. Wasmund, M. Nierychlo, Z. Kondrotaite, J. F. Petersen, M. Peces, M. S. Dueholm, M. Wagner, P. H. Nielsen

**Affiliations:** 1 Center for Microbial Communities, Department of Chemistry and Bioscience, Aalborg University, Aalborg, Denmark; 2 Division of Microbial Ecology, Centre for Microbiology and Environmental Systems Science, University of Vienna, Vienna, Austria

**Keywords:** Water microbiology, Applied microbiology

## Abstract

The bacterial genus *Tetrasphaera* encompasses abundant polyphosphate accumulating organisms (PAOs) that are responsible for enhanced biological phosphorus removal (EBPR) in wastewater treatment plants. Recent analyses of genomes from pure cultures revealed that 16S rRNA genes cannot resolve the lineage, and that *Tetrasphaera* spp. are from several different genera within the *Dermatophilaceae*. Here, we examine 14 recently recovered high-quality metagenome-assembled genomes from wastewater treatment plants containing full-length 16S rRNA genes identified as *Tetrasphaera*, 11 of which belong to the uncultured *Tetrasphaera* clade 3. We find that this clade represents two distinct genera, named here *Ca*. Phosphoribacter and *Ca*. Lutibacillus, and reveal that the widely used model organism *Tetrasphaera elongata* is less relevant for physiological predictions of this uncultured group. *Ca*. Phosphoribacter incorporates species diversity unresolved at the 16S rRNA gene level, with the two most abundant and often co-occurring species encoding identical V1-V3 16S rRNA gene amplicon sequence variants but different metabolic capabilities, and possibly, niches. Both *Ca*. P. hodrii and *Ca*. P. baldrii were visualised using fluorescence in situ hybridisation (FISH), and PAO capabilities were confirmed with FISH-Raman microspectroscopy and phosphate cycling experiments. *Ca*. Phosphoribacter represents the most abundant former *Tetrasphaera* lineage and PAO in EPBR systems in Denmark and globally.

## Introduction

Phosphorus is integral to manufacturing and the agricultural industry due to its requirement in household products, fertilisers, and animal feeds [1]. However, global reserves of phosphate rock are limited, making phosphorus a priority for sustainability initiatives and resource recovery [2]. Enhanced biological phosphorus removal (EBPR) is an economically attractive and microbially based method practised by wastewater treatment plants (WWTPs) worldwide to remove and (sometimes) recover phosphorus from wastewater. This process leverages unique features of the metabolisms of polyphosphate-accumulating organisms (PAOs) during anaerobic and aerobic cycling [3]. PAOs have been extensively studied over the past 50 years [4], and while *Candidatus* Accumulibacter (Proteobacteria) has long been considered to be the key PAO worldwide [3, 5], the genus *Tetrasphaera* (Actinobacteriota) has recently been determined to have equal, if not greater functional importance to many EBPR systems [4, 6]. In fact, *Tetrasphaera* spp. are frequently the most abundant PAOs in full-scale EBPR systems both in Denmark and globally [4].

*Tetrasphaera* spp. were identified as putative PAOs in 2000 [7], and since then the following eight species have been isolated and described: *T. elongata*, *T. australiensis*, *T. japonica*, *T. duodecadis*, *T. jenkinsii*, *T. vanveenii*, *T. veronensis* and *T. remsis* [8]. The classical PAO phenotype model has been inferred from properties of *Ca*. Accumulibacter and involves the accumulation of polyhydroxyalkanoates (PHA) from volatile fatty acids (VFAs) and glycogen during anoxic conditions [9]. Under oxic conditions where VFAs are scarce, PHA is used as an energy source, driving the accumulation of polyphosphate (polyP), glycogen formation and cell growth [9, 10]. *Tetrasphaera* spp. do not follow this classical PAO model as most species lack PHA storage capabilities, and the genus appears to have a more versatile metabolism than *Ca*. Accumulibacter [11]. In *T. elongata*, the most extensively studied *Tetrasphaera* sp., polyP is stored intracellularly under oxic conditions and released under anoxic conditions. During anoxic conditions, the organic substrates to support polyP cycling were thought to be amino acids and glucose that were stored as free amino acids and glycogen, respectively [10, 12]. However, as glycogen was not detected in *T. elongata* or *Tetrasphaera* in situ in activated sludge [6], its role is uncertain.

Historically, phylogenetic classification of the genus *Tetrasphaera* has been based on the 16S rRNA gene, which divided the genus into three clades [11]. Clade 1 and 2 incorporate the isolates *T. elongata* and *T. jenkinsii* (among others), respectively, whereas clade 3 incorporates only environmental sequences. So far, clade 3 has only been described via in situ studies [11–13]. Genome-based phylogeny recently showed that isolated members of the genus *Tetrasphaera* rather belong to three genera within the *Intrasporangiaceae*: *Phycicoccus* (*T. elongata*), *Knoellia* (*T. remsis*), and *Tetrasphaera* (*T. australiensis*), with *T. japonica* as an outgroup [14, 15]. Complete analysis of the bacterial genome tree has further refined these groups, reclassifying their parent family as *Dermatophilaceae*, and supporting the split of *T. japonica* from *T. australiensis* and *T. jenkinsii* [16, 17]. To date, no isolate or metagenome-assembled genome (MAG) has been recovered for clade 3 of the paraphyletic lineage. Obtaining genomes or isolates of former *Tetrasphaera* clade 3 is critical for understanding key PAOs, because it incorporates the dominant 16S rRNA gene-defined species across Danish WWTPs, known in MiDAS3 database as midas_s_5 [18].

Here, we investigate the most abundant PAOs from the former *Tetrasphaera* in Danish and global WWTPs, and show that they encompass two novel genera. Using 14 new high-quality MAGs and the recently released MiDAS4 global database [19, 20], we investigate their diversity and abundance in WWTP systems worldwide. We provide new insights into the niches, diversity, morphology and metabolic potential of the redefined lineages, which can be used to help inform future practices for phosphorus removal and recovery in the wastewater industry.

## Methods

### Phylogenetic analyses of the former Tetrasphaera genomes

*Tetrasphaera* MAGs were identified within the high-quality (HQ) MAGs recovered in Singleton et al. [19] using GTDB-Tk v1.4.1 [21] and the MiDAS3 [18] database (Supplementary Data File 1). Multiple sequence alignments of the concatenated 120 single copy proteins from GTDB-Tk, trimmed to 5000 amino acids, were input to IQ-TREE v2.0 [22] to create a maximum-likelihood tree using the WAG + G model, and bootstrapped 100×. “Tetrasphaera related cluster” (TRC) genomes were chosen based on the initial GTDB-Tk tree and the paraphyletic clade incorporating all former *Tetrasphaera* lineages (Supplementary Data File 2). The resulting bootstrapped tree of the TRC was examined in ARB v6.0.3 [23], and ITOL v5.7 [24] was used for tree visualisation with final aesthetic changes made in Inkscape v0.92. *Kineococcus rhizosphaerae*, *K. xinjiangensis* and *K. radiotolerans* were used as the outgroup.

The average nucleotide identity (ANI) between the MAGs and isolate genomes classified as belonging to the former *Tetrasphaera* was determined using a BLAST-based approach (ANIb) and pyani v0.2.10 [25], with the arguments ‘average_nucleotide_identity.py -m ANIb’. The resulting coverage and identity (ANIb_percentage_identity.tab) tables were processed in R v4.0.3 using the libraries gplots, RColorBrewer and reshape2, and heatmap.2 to produce the heatmaps (Supplementary Fig. 1 and Supplementary Data File 3). CheckM v1.1.2 [26] using the ‘lineage_wf’ was run on the TRC genomes to produce the completeness and contamination estimates.

### Phylogenetic analyses of the 16S rRNA and 23S rRNA genes

16S rRNA and 23S rRNA genes from *Kineococcus* isolates, *K. xinjiangensis* and *K. radiotolerans*, were used as the outgroups. Prokka v1.14 was used to annotate all TRC genomes, and the 16S rRNA genes and 23S rRNA genes were extracted from the ‘.ffn’ gene files using Fxtract v2.3 (github.com/ctSkennerton/fxtract). The extracted sequences were aligned using MAFFT v7.470 [27], and two poor-quality sequence alignments, due to fragmented genes, were removed from each alignment file before tree building (for 16S rRNA genes GCA_000955875.1 and GCF_000576595.1, and for 23S rRNA gene GCA_000955875.1). IQ-TREE was used to create a maximum-likelihood tree from the gene alignments using the model finder argument ‘-m MFP’. The chosen models were TIM3 + F + I + G4 and TIM3 + F + R4 for the 16S rRNA and 23S rRNA gene trees, respectively, and both trees were bootstrapped 100×.

MAG 16S rRNA genes were linked to MiDAS3 amplicon sequence variants (ASV) and full-length ASVs as in Singleton et al. [19]. 16S rRNA gene ANIs for the 14 MAGs were determined using pyani on the gene sequences, following the same arguments as the genome ANIb calculations above (Supplementary Data File 3).

### Relative abundances based on 16S rRNA gene amplicons and metagenomes

Amplicon data, based on primers targeting the V1-V3 region of 16S rRNA genes, from the MiDAS3 [18] (Danish WWTPs) and MiDAS4 [20] (global WWTPs) databases were used for the analysis of *Tetrasphaera* species and ASV abundances across WWTPs both in Denmark and worldwide. The Danish data comprised 712 samples from 20 nutrient-removal WWTPs with at least 13,500 reads per sample, and the global data comprised 847 samples from 438 WWTPs and four process types, with at least 10,000 reads per sample. Data were processed and heatmaps were produced using R v3.5.1 [28] and RStudio [29], using ampvis2 v2.4.9 [30] and ggplot2 v3.2.1 [31]. The abundances of the species representatives belonging to the former *Tetrasphaera* were calculated across 69 Danish WWTP metagenomes as in Singleton et al. [19].

### Comparative genomics

Metabolisms of the TRC MAGs and isolate genomes were annotated using EnrichM v0.5.0 (github.com/geronimp/enrichM) as described previously (Singleton et al. [19]) (Supplementary Data Files 4 and 5). EnrichM ‘classify’ was used to determine the KEGG modules with at least 80% completeness (Supplementary Data Files 6 and 7). EnrichM “enrichment” was used to determine the enrichment of (KEGG orthology numbers) KOs in the midas_s_5 group compared to the rest of the TRC genomes, as well as differences in metabolism between the *Ca*. P. baldrii and *Ca*. P. hodrii MAGs. To compare, investigate synteny, and cross-validate the EnrichM KO annotations, the 14 HQ Danish MAGs were uploaded to the MicroScope Microbial Genome Annotation & Analysis Platform [32]. The pan-genome feature of the MicroScope platform was used to compare the coding sequences of *Ca*. P. baldrii and *Ca*. P. hodrii at 50% amino acid identity and 80% alignment coverage and determine the number of core and species-specific genes (Supplementary Table 1). The genes specific to each species were explored within the surrounding gene context using the genome browser, and additional checks were conducted using BLASTP against the NCBI nr database [33].

For further comparisons of functional protein content among the *Ca*. P. baldrii and *Ca*. P. hodrii MAGs (*n* = 6), we performed ortho-group analysis of all proteins called from MAGE annotations using OrthoFinder (v2.5.2) [34], using default settings and the DIAMOND sequence search option. Protein sequences from ortho-groups that were present in all three MAGs of one species, but absent in all three MAGs of the other species, were then extracted and subjected to functional annotation to obtain “functional descriptions” using EggNOG-mapper [35] using default settings (Minimum hit e-value 0.001; Minimum hit bit-score 60; Percentage identity 40; Minimum % of query coverage 20; Minimum % of subject coverage 20) (Supplementary Data File 8). Counts of functional descriptions were then made to identify highly represented (≥5) functional proteins.

To identify potentially secreted peptidases, all protein sequences from the 14 novel MAGs were subjected to PSORTb 3.0 v3.0.6 [36], then protein sequences predicted to be ‘extracellular’ were collected and subjected to BLASTP using DIAMOND v2.0.4 [37] against the MEROPS database [38] with an e-value threshold of 10^−20^. The MEROPS ‘pepunit_3.lib’ (Peptidase Protein Sequences) database (version 12.3) was downloaded as of October 21, 2021. Predicted peptidases were then collected and subjected to PRED-TAT analysis using the ‘original model’ [39], in order to identify proteins with signal peptide sequences for export from the cytoplasm. Additionally, predicted peptidases were subject to eggNOG-mapper analysis using default settings (e-value threshold of 0.001) [35], in order to identify peptidases that mapped to COG proteins likely involved in cell wall/membrane/envelope biogenesis (COG category ‘M’), which were subsequently removed. The final ‘extracellular peptidases’ therefore include those with signal peptides that did not map to COG category M.

### Full-scale activated sludge batch experiments for P cycling

Batch experiments were conducted on fresh activated sludge to determine the polyP-content per cell of FISH-identified *Tetrasphaera*-related cells under anoxic and oxic conditions. Fresh samples were collected from a full-scale Danish WWTP (Aalborg West) and aerated for 30 min to exhaust most intracellular carbon source reserves. Sludge was then transferred to 200 ml serum bottles and sealed with a butyl septum and aluminium cap. A substrate solution comprising acetate, glucose, and casamino acids was added, with a final concentration of the three components of 500, 250, and 250 mg L^−1^, respectively, as is usual in P-release experiments with activated sludge [6, 40]. Ultrapure nitrogen was used to flush the headspace in each bottle to ensure anoxic conditions. The serum bottles were kept at room temperature (~22 °C) with shaking for 3 h. Samples for ortho-P analysis were taken every 20 min for the first hour of the experiment, and every 30 min during the remaining 2 h. Initial samples (0 h) and at the end of the experiment (3 h) were fixed for FISH-Raman analyses with a final concentration of 50% ethanol or 4% paraformaldehyde (PFA), as previously described [41], and stored at −20 °C until analysis.

### Phylogenetic analysis, FISH probe design and evaluation

Phylogenetic analysis of 16S rRNA gene sequences and design of FISH probes for the novel species were performed using the ARB software v.6.0.6 [23]. A phylogenetic tree was built based on comparative analysis of aligned 16S rRNA gene sequences, retrieved from the MiDAS 4.8 database [20], using the maximum-likelihood method and 1000× replicates bootstrap analysis. Coverage and specificity of the FISH probes were validated in silico with the MathFISH software [42] for predicted hybridisation efficiencies with target sequences. When needed, unlabelled competitor probes were designed to prevent hybridisation with non-target matches (Supplementary Table 2). All probes were purchased from Biomers (Ulm, Germany), labelled with cyanine-3 (Cy3), cyanine-5 (Cy5), Atto 488, Atto 532, Atto 565, Atto 594, Atto 633 and Dy-681 fluorochromes.

New FISH probes targeting the 23S rRNA of *Ca*. P. baldrii (Pbr1) and *Ca*. P. hodrii (Pbr2) were designed by extracting all 23S rRNA gene sequences from the 1083 MAGs from Danish WWTP [19] using RNAmmer v1.2 [43], aligning all sequences with Muscle (implemented in Mega6 v10.1.7), and manually identifying sequence stretches (15–30 bp) that contained mismatches between target and non-target sequences (Supplementary Table 2). Potential probes were further examined in silico for other potential off-target hits using TestProbe (SILVA) [44] with up to 2 mismatches allowed, and only probes with none or very few potential hits (<2), with up to 2 mismatches were allowed. Formamide concentrations required for stringent conditions were also predicted in silico using MathFISH [42].

### Fluorescence in situ hybridisation (FISH) and Raman microspectroscopy

FISH was performed as previously described, with study-specific modifications [45]. Raman microspectroscopy was applied in combination with FISH as previously described in order to detect intracellular polyP, glycogen and PHA [6] (see Supplementary Information for details).

## Results and discussion

### Former Tetrasphaera are paraphyletic and form at least five different genera

**Fig. 1:**
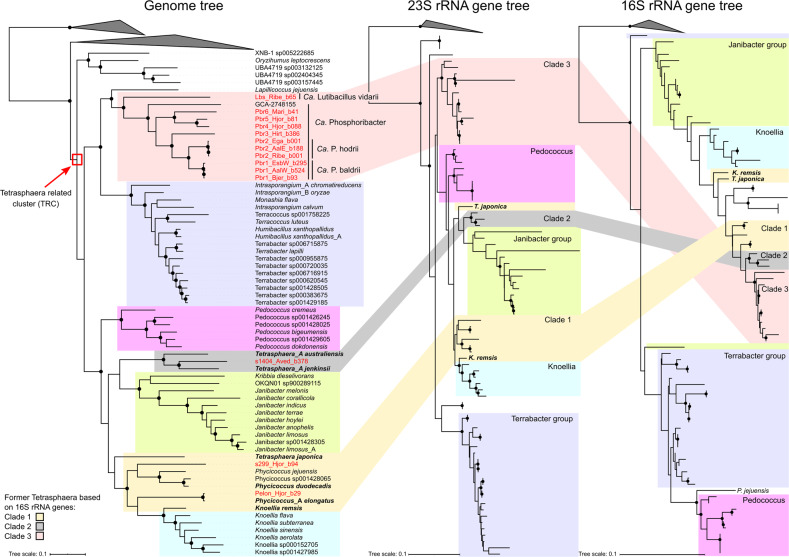
Comparisons of the genome tree vs 23S rRNA gene tree vs 16S rRNA gene tree (bootstrap support >70% is indicated by the black circles). The maximum-likelihood genome tree was created from the concatenated alignment of 120 single copy marker gene proteins trimmed to 5000 amino acids using GTDB-Tk and 100 bootstraps. The “_A” suffix, appended by GTDB-Tk, indicates genera that should likely be split into two. Some groups defined in the genome tree, such as *Janibacter* and *Terrabacter*, split in the 16S rRNA gene tree, which has poor bootstrap support. The maximum-likelihood rRNA gene trees were created from the alignment of the rRNA genes extracted from the genomes, and were resampled using 100× bootstraps. For NCBI GenBank genome accession numbers see Supplementary Data File 2. TRC: *Tetrasphaera*-related cluster”.

A set of 1083 high-quality MAGs recovered from Danish WWTPs was searched for MAGs encoding 16S rRNA genes belonging to the *Tetrasphaera* cluster, and 14 MAGs were identified [19]. Genome-based phylogenetic analysis of these MAGs supported recent findings that the “former *Tetrasphaera*” are paraphyletic within the *Dermatophilaceae* (Fig. 1) (Supplementary Data File 1) [14]. The isolate genomes are situated in different characterised genera, such as *Knoellia* and *Phycicoccus*, revealing the limitations of 16S rRNA gene-based phylogenies for this family. Strikingly, the genome-based phylogeny showed that 13 of the newly described MAGs are distinct from previously described *Tetrasphaera* and other members of the *Dermatophilaceae* (further refined below). Although dispersed throughout the family, the genomes followed the same clade distinctions as observed in the 16S rRNA genes: clades 1, 2 and 3 (Fig. 1). While isolates have been cultivated for clade 1 and 2 of the former *Tetrasphaera*, clade 3 is represented only by environmental 16S rRNA gene sequences.

Mapping of the 16S rRNA genes to the MiDAS3 full-length ASV reference database indicated the genomes represented members of the 16S rRNA gene-defined de novo species *Tetrasphaera* midas_s_5 (10 MAGs), midas_s_45 (1 MAG), midas_s_1404 (1 MAG), midas_s_299 (1 MAG) and the isolate *T. elongata* (1 MAG) [18, 46] (Supplementary Data File 1). The midas_s_1404 MAG grouped with *Tetrasphaera australiensis* (genome taxonomy database (GTDB) tax. *Tetrasphaera*_A australiensis) and *T. jenkinsii* in the genome tree, whereas midas_s_299 grouped with *Tetrasphaera duodecadis* (GTDB tax. *Phycicoccus*) and *P. jejeunsis*. The *T. elongata* MAG is nearly identical (>98% ANI) to *Tetrasphaera elongata* (GTDB tax. *Phycicoccus_A*), which clustered near *Tetrasphaera remsis* (GTDB tax. *Knoellia*). The split of genera, such as *Phycicoccus* and *Phycicoccus_A*, *Tetrasphaera* and *Tetrasphaera_A*, in GTDB [17] shows that the addition of genomes is improving the resolution of the family, but more genomes are needed to fully resolve these lineages (Table 1).

**Table 1:** Changing phylogenies of the former *Tetrasphaera*.

Original name or MiDAS name	GTDB RefSeq release 95 name	16S rRNA gene clade	Proposed species/placeholder name and ASV details
*Tetrasphaera japonica*	*Tetrasphaera japonica*	Clade 1	NA
*Tetrasphaera midas_s_299*	f__*Dermatophilaceae*	Clade 1	s299_Hjor_b94 (ASV51)
*Tetrasphaera duodecadis*	*Phycicoccus duodecadis*	Clade 1	NA
*Tetrasphaera elongata*	*Phycicoccus_A elongatus*	Clade 1	Pelon_Hjor_b29 (ASV70)
*Tetrasphaera remsis*	*Knoellia remsis*	Clade 1	NA
*Tetrasphaera australiensis*	*Tetrasphaera_A australiensis*	Clade 2	NA
*Tetrasphaera midas_s_1404*	*Tetrasphaera_A*	Clade 2	s1404_Aved_b378 (776)
*Tetrasphaera jenkinsii*	*Tetrasphaera_A jenkinsii*	Clade 2	NA
*Tetrasphaera* midas_s_5	g__GCA-2748155	Clade 3 - *Ca*. Phosphoribacter	*Ca*. P. baldrii (Pbr1, ASV1)
			*Ca*. P. hodrii (Pbr2, ASV1, ASV84913, ASV22244)
			Pbr3 (ASV22244)
			Pbr4 (ASV12)
			Pbr5 (ASV55)
			Pbr6 (ASV12)
*Tetrasphaera* midas_s_45	g__GCA-2748155	Clade 3 - *Ca*. Lutibacillus	*Ca*. L. vidarii (Lbs, ASV15)

The original name is shown, as well as the GTDB classification, the 16S rRNA gene clade, and the names used and proposed in this study. The GTDB classification g__GCA-2748155 indicates a placeholder genus name based on the medium quality MAG, and midas_s_1404 has no species association, and midas_s_299 has no GTDB genus or species association. Pbr = *Ca*. Phosphoribacter, with numbers indicating the distinct species (e.g. Pbr1-6). Lbs = *Ca*. Lutibacillus. The GTDB names and proposed names are used in this study. NA: Not available.

Both midas_s_5 and midas_s_45 belonged to the 16S rRNA gene clade 3, the most abundant yet uncultivated clade in WWTPs worldwide [20]. The 11 midas_s_5 and midas_s_45 MAGs clustered with only one medium quality MAG (77.8% complete) in GTDB (RefSeq release 95). MAG GCA_2748155 belongs to a currently uncharacterised genus and was recovered from a dolphin oral metagenome [47], suggesting the lineage is not limited to WWTPs. This clade could be subdivided further into two genera using full genome ANI and the proposed genus ANI boundary of 75–77% [17, 48] (Supplementary Fig. 1). This revealed that the midas_s_45 MAG likely belongs to one novel genus, and midas_s_5 MAGs to another, named here *Ca*. Lutibacillus and *Ca*. Phosphoribacter, respectively (Table 1). Furthermore, despite the high sequence identity between 16S rRNA gene sequences in *Ca*. Phosphoribacter (97.53–100%) (Supplementary Data File 2), this lineage represents six distinct species (Pbr1-6) based on genome ANI (<95% similarity), again highlighting the lack of resolution provided by 16S rRNA genes in this group.

While the 16S rRNA gene tree and genome trees had discordant phylogenies, the 23S rRNA gene tree was largely concordant with the genome tree for all clades (Fig. 1). The most prominent issue with a 16S rRNA gene-based phylogeny for this group was the high similarity of the 16S rRNA gene sequences, as the 14 MAGs had ANIs between 95.55% and 100% (Supplementary Data File 2), which is problematic considering 98.7% and 94.5% are the standard species and genus boundaries for 16S rRNA gene sequences, respectively [49]. In general, discrepancies between rRNA gene trees and genome trees are common in the Actinobacteriota [14], highlighting the need for high-quality genomes with full-length 16S rRNA genes when investigating lineages of interest. Based on the phylogenetic analyses, members of the former *Tetrasphaera* genus likely belong to 6–7 different genera.

### Geographical distribution of the redefined Tetrasphaera lineages in WWTP

**Fig. 2:**
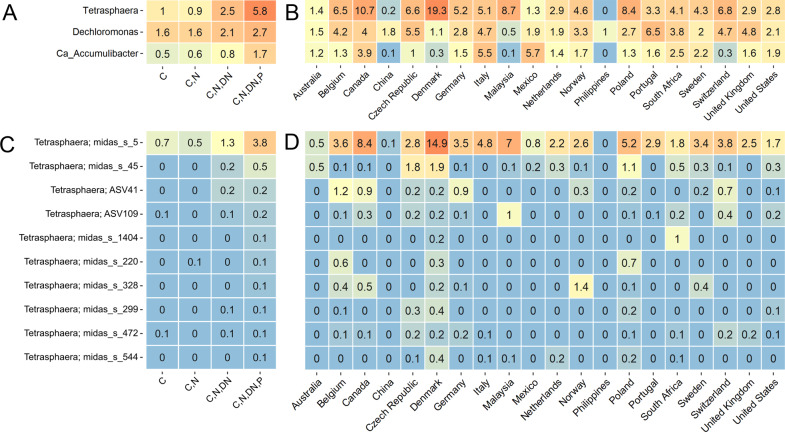
Global average percent relative abundance of the most abundant known PAOs (genera). Abundance is shown across (**A**) different process configurations (N plants = 480; C - carbon removal; C,N - carbon removal and nitrification; C,N,DN - carbon removal, nitrification and denitrification; C,N,DN,P - carbon removal, nitrification, denitrification and phosphorus removal), and (**B**) across the world in plants with N and P-removal (C,N,DN,P) plants (N plants = 111). The same is shown for *Tetrasphaera* species in **C** and (**D**) Taxa without species classification are shown as individual ASVs. Data comes from the global survey of microbial communities in WWTPs (Dueholm et al. [20]).

Populations belonging to *Ca*. Accumulibacter and the newly described *Dechloromonas* [50] are, along with bacteria previously ascribed to genus *Tetrasphaera*, the most prevalent known PAOs in Danish and global WWTPs with EBPR (Fig. 2A, B). *Tetrasphaera* was generally the most prevalent genus, and had bacterial species with average relative abundances per plant often exceeding 10% of all bacteria based on 16S rRNA gene sequencing (Fig. 2B, Supplementary Fig. 2) [18]. We investigated the fine-scale distribution of these lineages based on 16S rRNA gene V1-V3 variable region ASV data from the MiDAS databases both within Denmark and globally (Table 1, Supplementary Data File 1). Six of the seven most abundant *Tetrasphaera* ASV lineages in Danish WWTPs were represented by at least one of the newly recovered MAGs (Supplementary Figure 3). *Tetrasphaera* midas_s_5 ASV1 (represented by 5 MAGs) was consistently the most abundant of the ASV lineages, followed by midas_s_220 ASV7, midas_s_5 ASV12 (2 MAGs), and midas_s_45 ASV15 (1 MAG) (Supplementary Fig. 3). Other than midas_s_5 ASV1, the most abundant ASV lineages averaged across the globe differed from the Danish averages (Supplementary Fig. 4). After clustering at the species level, midas_s_5, midas_s_45, midas_s_299 and midas_s_220 were among the top 10 most abundant species both in Denmark and globally (Supplementary Fig. 3; Fig. 2D). The highest average relative abundances of the 16S rRNA defined *Tetrasphaera* in EBPR WWTPs were found in Denmark (14.9%), Canada (8.4%), and Malaysia (7%). In contrast, minimal abundances (≤0.1%) were recorded for several countries, these being China, Mexico, and Australia (Fig. 2B). The distribution of *Tetrasphaera* was associated with specific activated sludge process types, as abundances were higher in EBPR plants (C,N,DN,P, i.e., carbon, nitrogen and phosphorus removal) compared to simple plants with only carbon removal or with carbon removal and nitrification (C and C,N) (Fig. 2C).

Based on the genomic approaches (Fig. 1), midas_s_5 ASV1 was found to represent two separate species, named here *Ca*. Phosphoribacter baldrii (Pbr1) and *Ca*. P. hodrii (Pbr2), despite having identical 16S rRNA gene sequences across the 300 bp amplicons (and >99% identity over their full-length 16S rRNA genes) (Supplementary Data File 2). In order to investigate this lineage at species-level resolution, we mapped 69 metagenomes from 23 Danish WWTPs to the 10 MAG species representatives using stringent alignment and identity cut-offs (Supplementary Fig. 5). Multiple species were detected in most WWTP metagenomes, with the WWTP Hjørring having the highest diversity and abundance. High abundances of *Ca*. Phosphoribacter and *Ca*. Lutibacillus lineages supported the 16S rRNA gene amplicon analysis. *Ca*. P. baldrii and *Ca*. P. hodrii appeared together and in high abundances across the metagenomes (Supplementary Fig. 5), suggesting that their ecological niches do not directly overlap (see below).

### Comparative genomics and metabolic potential of the redefined genera

**Fig. 3:**
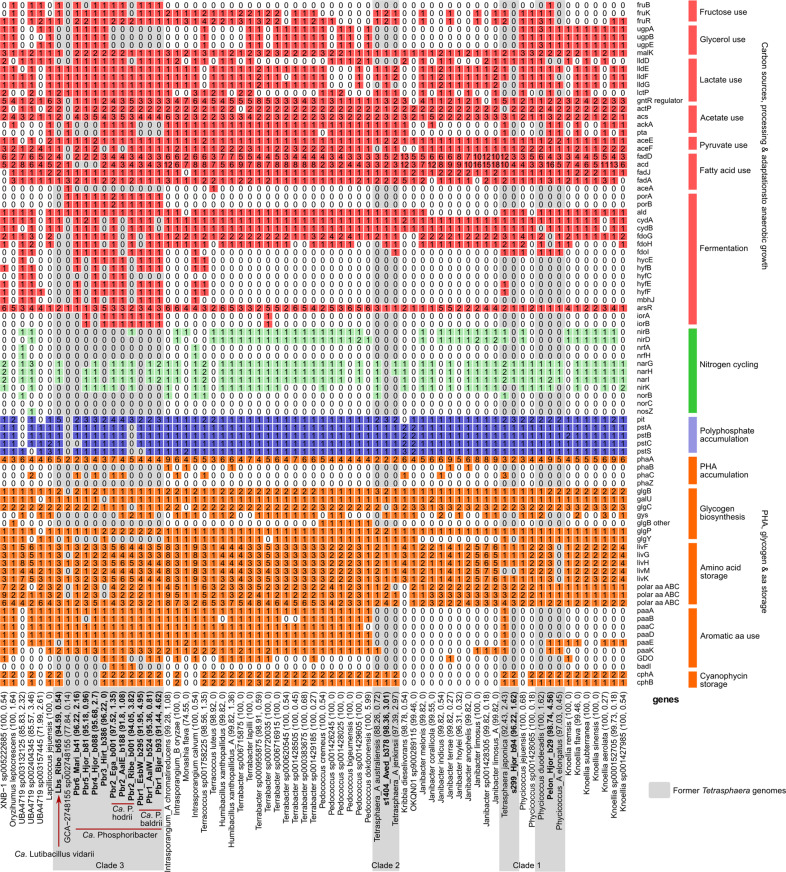
Basic functional potential of the *Tetrasphaera* MAGs and closest relatives (*Tetrasphaera* related cluster, TRC), with focus on functions related to nutrient-removal physiologies. The gene list follows the progression in the text. For the full list of gene names and associated KO numbers see Supplementary Data File 4. The MAGs and genomes are ordered by the genome tree in Fig. 1, with their genome completeness and contamination indicated within the parentheses. Numbers in the plot represent the number of protein sequences identified in each MAG by EnrichM (i.e., number of hits to KOs).

The former *Tetrasphaera* lineage comprises multiple genera, consequently many of its members, including the abundant *Ca*. Phosphoribacter and *Ca*. Lutibacillus clade 3 groups, could have metabolisms that cannot be reliably described by the model isolate *P. elongatus*. As the three clades of the former *Tetrasphaera* are spread throughout the *Dermatophilaceae* and among microorganisms from a variety of environments, including human pathogens such as *Janibacter melonis* [51], we investigated specific metabolic traits, especially those potentially relevant to nutrient-removal physiologies, of the paraphyletic group referred to here as the “*Tetrasphaera*-related cluster” (TRC; Figs. 1 and 3). We aimed to determine how clade 3 differs in metabolic potential to the broader TRC, including the other former *Tetrasphaera* isolates, and to create a new metabolic model describing the two most abundant species of clade 3.

All three of the former *Tetrasphaera* clades encoded complete central carbon processing pathways: the pentose phosphate pathway, glycolysis, and the TCA cycle. A wide selection of carbon sources are used by the lineages, as indicated by the genomic potential (Supplementary Note 1). Notably, previous isolates have shown growth on glucose [10], and putative ABC transporters were identified that could facilitate import of a range of simple sugars such as glucose and xylose [52]. For *Ca*. Phosphoribacter, fructose and glycerol-3-phosphate use seemed to be enriched compared to the other TRC (Fig. 3) [53].

*P. elongatus* was found to use lactate aerobically as a carbon source [54]. Use of lactate as a carbon source by the clade 3 group and wider TRC is similarly indicated by the presence of genes for lactate utilisation (Supplementary Note 1). The potential for beta-oxidation, or due to shared enzymes, isoleucine, valine or leucine degradation, was also widely distributed across the TRC (Supplementary Note 1). However, the glyoxylate cycle was complete (with *aceA* isocitrate lyase) in only two genomes, suggesting limited fatty acid catabolism in the TRC [55].

Fermentation of substrates to acetate, lactate, alanine and succinate has been determined in the former *Tetrasphaera* isolates either through experimental measurements or based on genomic potential [10]. All former clade 3 and nearly all TRC MAGs (58/69) encoded the alanine dehydrogenase (*ald*) for the oxidation of alanine to pyruvate. However, only the *Ca*. P. hodrii and Pbr3 MAGs encoded the full fermentation to acetate pathway, which is missing in the other *Ca*. Phosphoribacter MAGs (missing *pta* and *ackA*) (Fig. 3; Supplementary Note 1). Genes for pyruvate ferredoxin oxidoreductases (*porA*, *porB*) were present in 10/11 clade 3 MAGs, i.e., all *Ca*. Phosphoribacter MAGs. Additionally, indolepyruvate oxidoreductases (*iorA*, *iorB*) were also present in 8/11 clade 3 MAGs. Strikingly, both *porAB* and *iorAB* were absent from *Ca*. Lutibacter and nearly all other TRC (Fig. 3). Both Por and Ior are typically oxygen-sensitive enzymes that may be used during anaerobic or microoxic aromatic amino acid fermentation [56]. These results therefore indicate a potentially key physiological difference among *Ca*. Phosphoribacter and other members of the *Dermatophilaceae*.

A respiratory nitrate reductase (NarGHI) was encoded in all former *Tetrasphaera* isolates except *K. remsis* and *T. jenkinsii*. Similarly, most TRC MAGs from the Danish WWTPs also encoded enzymes for nitrate reduction, with the exceptions being MAGs s1404_Aved_b378, Pbr1_EsbW_b295, Pbr3 and Pbr5. Genes for nitrite, nitric oxide and nitrous oxide reduction were less widespread (Supplementary Note 1).

We examined the prevalence of genes important for, but not limited to, polyphosphate accumulation and storage. These genes were identified widely across the TRC (Fig. 3). Nearly all MAGs encoded the low-affinity phosphate transporter Pit, suggested to be the best genetic indicator of a possible PAO phenotype [57], and the high-affinity phosphate transporter encoded by *pstSCAB* (Fig. 3). Other genes associated with polyphosphate metabolism were nearly ubiquitous. Polyphosphate kinase (*ppk*) and exopolyphosphatase (*ppx-gppA*) were encoded in all TRC genomes, similarly adenylate kinase (*adk*) was encoded in nearly all TRC genomes (67/69) (Supplementary Data File 4). At the genomic level, polyphosphate accumulation appears possible for many members of the *Dermatophilaceae*, but the environmental conditions likely determine the storage and cycling phenotype, and as always, experimental evidence is required for confirmation of this metabolic trait (see below).

Storage compounds, such as glycogen and PHA, are believed to be integral to the PAO phenotype by providing energy for polyP accumulation during oxic conditions and production of PHA under anoxic conditions [10]. None of the TRC genomes encoded all three enzymes for PHA synthesis (i.e., PhaABC, Fig. 3). Two *Ca*. P. hodrii MAGs and *T. japonica* encoded PhaA and PhaC, and PHA has been detected using gas chromatography in *T. japonica* [10], suggesting that *Ca*. P. hodrii may also be capable of PHA storage, however no PHA was detected experimentally (see below). Previous genome studies predicted *Tetrasphaera* produced glycogen as an energy storage compound [10]. However, recent work using probe Actino658, which we now know targets *Ca*. P. baldrii, *Ca*. P. hodrii and Pbr3 (see below), showed glycogen was not detectable in individual FISH-defined *Tetrasphaera* cells by Raman microspectroscopy in activated sludge samples from EBPR plants [6]. Some genes for glycogen synthesis were identified in the TRC genomes (Fig. 3), however we propose that the TRC may synthesise glycogen-like α-glucan polysaccharides as cell-wall capsular material, similar to other Gram-positive Actinobacteriota, rather than glycogen for storage (Supplementary Note 1).

*P. elongatus* is believed to be capable of accumulation of amino acids under anoxic conditions, which are used as an energy source to ‘take up‘ phosphate during oxic conditions [12]. We explored the presence of amino acid transporter genes in order to investigate if amino acids could also be important energy sources for the clade 3 lineages. Amino acids such as lysine, arginine, histidine, leucine, isoleucine, valine and phenylalanine are likely important growth substrates based on the identified transporters, and the presence of genes for aromatic acid catabolism were also enriched in the clade 3 MAGs (Fig. 3, Supplementary Note 1). Among the novel clade 3 MAGs, various peptidases (ranging from 2 to 7 per MAG) that are predicted to be secreted to the extracellular environment were present among all MAGs except (s5c6_Mari_b41), suggesting they can actively digest extracellular proteins/peptides for acquiring amino acids (Supplementary Data File 8). Predicted secreted peptidases were also common among the TRC genomes, ranging from 1 to 19 per genome (Supplementary Data File 9). Interestingly, while uncommon in bacteria, Actinobacteriota often encode and make use of proteasomes for degrading proteins [58], and 63/69 TRC genomes encoded them (Supplementary Data Files 4 and 6). Proteasomes could give the clade 3 lineages an advantage over *Ca*. Accumulibacter, enabling them to recycle resources from proteins and potentially respond quickly to challenging and fluctuating conditions [58], such as in WWTPs. MAGs from *Ca*. Accumulibacter lacked predicted secreted peptidases.

An alternative energy storage compound could be cyanophycin, because genes for cyanophycin synthetase (*cphA*) and cyanophycinase (*cphB*) were identified in 48 of the 69 TRC genomes, including all former *Tetrasphaera* MAGs and isolates, except for some MAGs affiliated with *Ca*. Phosphoribacter (Pbr4-6) (Fig. 3). Most commonly found in Cyanobacteria, cyanophycin is a polymer of arginine and aspartic acid, and therefore a nitrogen (and carbon) storage compound [59–61].

### Differences between the most abundant species Ca. P. badrii and hodrii

**Fig. 4:**
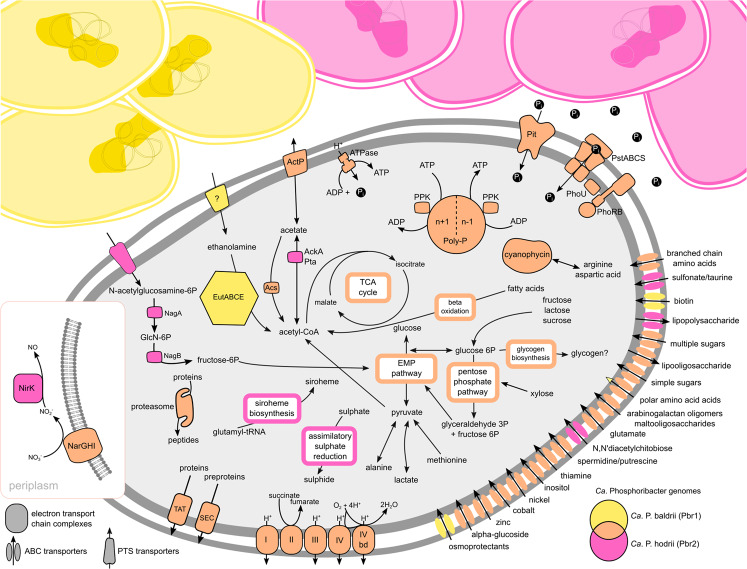
Metabolic model of *Ca*. Phosphoribacter baldrii and *Ca*. P. hodrii. Colours indicate the presence of genes in either both (orange) or only one (yellow = *Ca*. P. baldrii, pink = *Ca*. P. hodrii) species. For specific genes of pathways see Supplementary Data Files 4, 6 and 7. Abbreviations: EMP pathway, Embden–Meyerhof–Parnas pathway (glycolysis); TCA cycle, tricarboxylic acid cycle; electron transport chain (I, complex I NADH dehydrogenase; II, complex II succinate dehydrogenase; III, complex III cytochrome bc1; IV, cytochrome *c* oxidase; IV, cytochrome *bd* oxidase); nitrate reductase respiratory (NarGHI), nitrite reductase (NirK), TAT and SEC dedicated protein secretion, inorganic phosphate transporter family (Pit), inorganic phosphate ABC transporter (PstABCS), phosphate transport system accessory protein (PhoU), two-component system for phosphate regulation (PhoRB), polyphosphate (Poly-P), polyphosphate kinase (PPK), acetate kinase (AckA), phosphotransacetylase (Pta), acetyl-CoA synthetase (Acs), N-acetylglucosamine-6-phosphate deacetylase (NagA), glucosamine-6-phosphate deaminase (NagB), ethanolamine utilisation protein (EutA), ethanolamine ammonia-lyase (EutBC), acetaldehyde dehydrogenase (EutE). Question marks indicate unknown uses or transporters.

The top two most abundant former *Tetrasphaera* species, *Ca*. Phosphoribacter baldrii (Pbr1, 3 MAGs) and *Ca*. Phosphoribacter hodrii (Pbr2, 3 MAGs) share the same 16S rRNA gene V1-V3 ASV (ASV1), but have only ~82% genome ANI and share a total of 1594 core-genome gene families and species-specific variable genomes of 1806 and 2156 gene families, respectively (Supplementary Fig. 1; Supplementary Table 1). These two populations occurred in high abundances in the same WWTP samples (Supplementary Fig. 5), suggesting that they do not occupy the exact same niches and that some of the metabolic differences may facilitate their reoccuring coexistence (Fig. 4). To examine potential functional differences among the two dominant *Ca*. Phosphoribacter species, we performed ortho-group (OG) analysis of protein sequences from the six MAGs in order to compare OGs that were unique to each species. For this, we specifically compared proteins that were present in all three MAGs of each species, but were absent in the three MAGs of the other species (Supplementary Data File 8). This identified 371 OGs (consisting of 1129 proteins) unique to *Ca*. P. baldrii, and 355 OGs (consisting of 1070 proteins) unique to *Ca*. P. hodrii (Supplementary Data File 8). We then obtained functional descriptive annotations of proteins that were unique to each species, and specifically describe those with numerous (≥5) proteins (Supplementary Data File 8). We propose these may represent especially important groups of proteins because of their abundances (all other proteins of OGs unique to either species are also presented in Supplementary Data File 8). This revealed that *Ca*. P. baldrii was enriched in unique genes for transposases, various probable virulence/effector factor-like proteins, toxin/antitoxin (type II) proteins, as well as biotin and folate import and modification proteins. *Ca*. P. hodrii was enriched in unique genes for use of sugars, e.g., gluco- and fructo-kinases, sucrose use (sucrose-6-phosphate hydrolase), and cytochrome-P450 (co-factor of monoxygenases), among other proteins likely involved in biosyntheses of cell wall components and co-factors. Both species also encoded various unique proteins related to transporters and/or porins, various transcriptional regulators, proteins potentially involved in antibiotic resistance, as well as various proteins with unknown functions. Of particular interest for understanding nutrient acquisition, we identified that *Ca*. P. baldrii (and *Ca*. Lutibacillus vidarii) have the potential to use ethanolamine, which is abundant in many bacterial and mammalian cells as part of the lipid phosphatidylethanolamine [62]. Intriguingly, phosphotidylethanolamine is broken-down to glycerol and ethanolamine, and *Ca*. P. baldrii has genes to use both glycerol and ethanolamine, while *Ca*. P hodrii does not. Although concentrations in wastewater are unknown, we propose it is likely readily available in activated sludge systems as a component of residual faecal matter in influent because it is abundant in faecal matter [63, 64], and/or from bacterial necromass turn-over in the system [62] (Supplementary Note 2). *Ca*. P. hodrii encoded the potential to use the sugar N-acetylglucosamine as a carbon and nitrogen source [65], similar to *T. remsis* [66] (Supplementary Note 2). N-acetylglucosamine is a common component of biological polymers, such as chitin and cell walls [65], and like ethanolamine would be readily available in activated sludge systems. This species also encoded the potential for aerobic acetate production from acetyl-CoA, acetate uptake, or fermentation of pyruvate to acetate via the *pta* and *ackA* genes, both of which were missing in the *Ca*. P. baldrii MAGs (Fig. 4).

### Visualisation and experimental confirmation of the storage polymers of the redefined genera

**Fig. 5:**
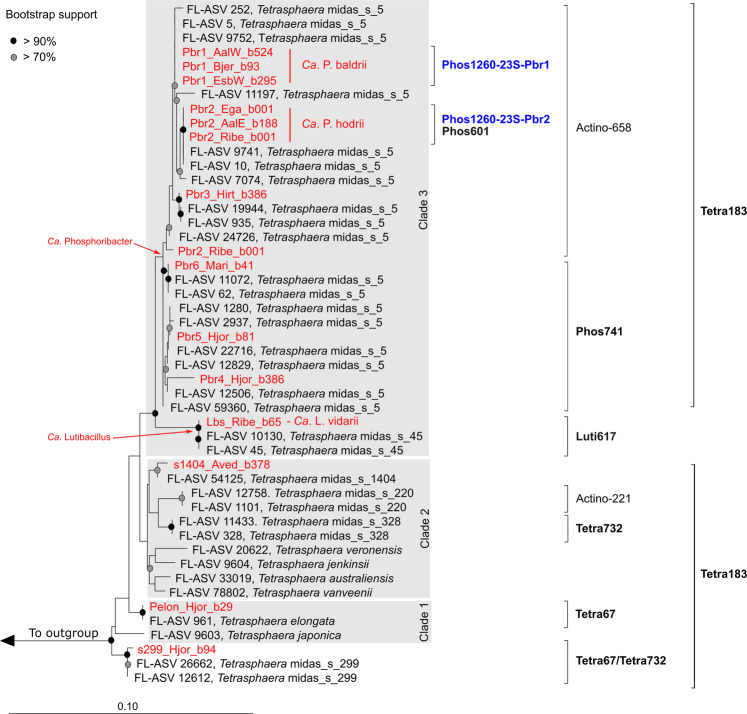
Maximum-likelihood (PHYML) 16S rRNA gene phylogenetic tree of “*Tetrasphaera”* species abundant in Danish and global activated sludge plants. Leaves indicated in red represent 16S rRNA gene sequences retrieved from the MAGs, leaves in black represent entries from the MiDAS4.8 database. Coverage of FISH probes is shown by black brackets (in bold - probes designed in this study). The probe indicated by the blue colour is targeting 23S rRNA. The grey boxes indicate the clade classification. MiDAS4.8 sequences belonging to the families *Intrasporangiaceae* and *Dermatophilaceae* were used as an outgroup. The scale bar represents substitutions per nucleotide base.

**Fig. 6:**
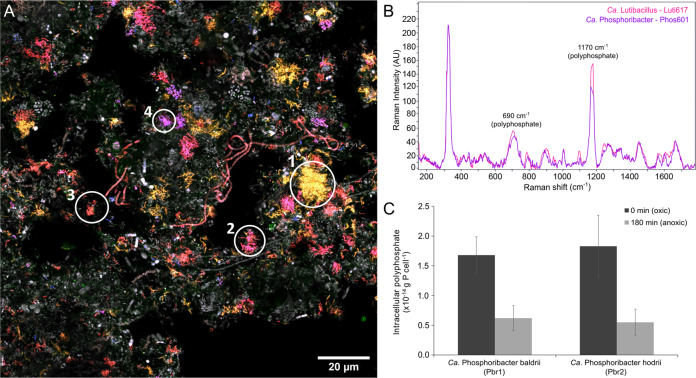
Tetrasphaera-related genera ecophysiology in activated sludge. **A** Multicolour FISH micrograph of different *Tetrasphaera*-related genera in full-scale activated sludge. *Ca*. Phosphoribacter baldrii (yellow; no.1) was targeted by Phos1260-23S-Pbr1 probe (Atto 488). *Ca*. Phosphoribacter hodrii (pink; no. 2) was targeted by Phos601 probe (Dy-681). Most of *Tetrasphaera*-related genera (orange; no. 3) were targeted with Tetra183 probe (Atto 532). *Ca*. Lutibacillus (purple; no. 4) was targeted by Luti617 probe (Atto 594). All bacteria (grey) were targeted with EUBmix probe (Atto 633). **B** Raman spectra of *Ca*. Lutibacillus and *Ca*. Phosphoribacter cells containing large amounts of polyphosphate. **C** Dynamics of poly-P in FISH-defined cells for the two most abundant species, *Ca*. P. baldrii and hodrii, during aerobic/anaerobic alternating phases, 100 cells were measured for each species.

We designed new FISH probes to specifically visualise and aid experimental confirmation of polyphosphate accumulation and other storage compounds in the novel clade 3 lineages *Ca*. Phosphoribacter and *Ca*. Lutibacillus (Table 2) (Supplementary Note 3). Species-specific probes were developed for the three most abundant lineages in Danish WWTPs, i.e., *Ca*. Phosphoribacter baldrii (Pbr1), *Ca*. P. hodrii (Pbr2) and *Ca*. Lutibacillus vidarii (Lbs) (Supplementary Table 2). Due to the similarity between *Ca*. P. baldrii and *Ca*. P. hodrii based on the 16S rRNA (Fig. 5), species-specific probes were also created targeting the 23S rRNA. An additional higher-level probe was created for Pbr4-6 (Figs. 1 and 5), and the previously published probe Actino658 covered *Ca*. P. baldrii, *Ca*. P. hodrii and Pbr3 [13]. All probes targeting lineages within the clade 3 group hybridised to rod-shaped cells of similar sizes (0.6–0.9 × 1–1.5 µm) and arranged in microcolonies embedded in the structure of the activated sludge floc (Fig. 6A, Supplementary Fig. 6).

**Table 2:** FISH probes, morphology and storage polymers of the former *Tetrasphaera* species.

Target	MAG name	Probe	Morphology (µm)			Storage polymers^a^		
			Coccus (diameter)	Rods (diameter × length)	Filament (diameter × length)	Poly-P	PHA	Glycogen
midas_s_220	–	Actino221	0.8–0.9	–	–	+	–	–
midas_s_5 (Pbr1, Pbr2, Pbr3)	*Ca*. P. baldrii, *Ca*. P. hodrii, Pbr3	Actino658	–	0.4–0.7 × 1–1.7	–	+	–	–
midas_s_5 (Pbr4, Pbr5, Pbr6)	Pbr4, Pbr5, Pbr6	Phos741	–	0.4–0.7 × 1–1.7	–	+	–	–
midas_s_5 (Pbr2)	*Ca*. P. hodrii	Phos601	–	0.4–0.7 × 1–1.7	–	+	–	–
midas_s_5 (Pbr1)	*Ca*. P. baldrii	Phos1260-23S-Pbr1	–	0.4–0.7 × 1–1.7	–	+	–	–
midas_s_5 (Pbr2)	*Ca*. P. hodrii	Phos1260-23S-Pbr2	–	0.4–0.7 × 1–1.7	–	+	–	–
midas_s_45	*Ca*. Lutibacillus	Luti617	0.6–0.8	0.5–0.89 × 2–4	–	+	–	–
midas_s_299, midas_s_328, midas_s_1378	–	Tetra732	–	0.5–0.6 × 1.1–1.2	1.2–1.3 × >100	+	–	–
midas_s_299, midas_s_469, *T. elongata*, midas_s_24955, midas_s_24809, midas_s_35051, midas_s_5540, midas_s_31199	–	Tetra67	–	0.5–0.6 × 1.1–1.2	0.6–0.7 × 20–90	+	–	–

^a^Detected by Raman microspectroscopy.

Application of the FISH probes in combination with Raman microspectroscopy on in situ cells from activated sludge revealed the presence of polyphosphate in all targeted species, but no other storage compounds (PHA or glycogen) were detected, which was in agreement with previous observations (Table 2) [6, 40]. The amount of polyphosphate stored within the cells was similar for *Ca*. Phosphoribacter and *Ca*. Lutibacillus (Fig. 6B). In order to quantify and explore the dynamics of polyphosphate in *Ca*. Phosphoribacter, we performed anaerobic-aerobic P-cycling experiments with fresh activated sludge from a full-scale EBPR plant (Supplementary Fig. 7). A mixture of carbon sources (acetate, glucose, and casamino acids) was added during the anaerobic phase as *Ca*. Phosphoribacter is suggested to use amino acids or sugars as substrates for P release under anoxic conditions [6, 67]. In situ quantification of polyphosphate was performed for the two most abundant species, *Ca*. P. baldrii and *Ca*. P. hodrii. Both species exhibited dynamic cycling of intracellular polyphosphate (Fig. 6C). They were as important as other PAOs in the sample, *Ca*. Accumulibacter and *Dechloromonas* (Supplementary Fig. 7B). This shows that both *Ca*. Phosphoribacter and *Ca*. Lutibacillus are important contributors to polyphosphate accumulation in the activated sludge system.

### Ecology of former Tetrasphaera and the importance for full-scale WWTP performance

Previously, it was widely believed that bacteria belonging to a clade within the genus *Tetrasphaera* were the most abundant PAOs in Danish and global WWTPs. Our phylogenomic analyses show that this clade 3 lineage actually comprises two globally abundant novel genera, and that the dominant species based on the 16S rRNA gene represents six distinct species at the genome level. Characterised isolates, previously assigned to the genus *Tetrasphaera*, belong to several different genera within the *Dermatophilaceae* and consequently cannot be used as models for the clade 3 lineages abundant in WWTPs, i.e., *Ca*. Phosphoribacter and *Ca*. Lutibacillus.

EBPR systems rely heavily on PAOs for phosphorus removal and encourage PAO growth through the use of aerobic and anaerobic cycling [68]. The distribution of the clade 3 genera, *Ca*. Phosphoribacter and *Ca*. Lutibacillus, is strongly linked to process designs with nitrogen and phosphorus removal, but not limited by geography. Besides process design, additional influencing factors affecting abundance are operational parameters and perhaps the amount of industrial wastewater [20]. However, recent research has also shown that immigration of microbial populations via the influent may be the determining factor for both presence and abundance of the clade 3 lineages within WWTP systems [69].

The clade 3 lineages have versatile metabolisms, with genomic potential indicating a likely capacity for use of various carbon sources, sugars and amino acids, under both oxic and anoxic conditions. Nitrate or nitrite could serve as electron acceptors for many of the clade 3 populations represented by the MAGs, suggesting that these groups are also involved in nitrogen cycling but not full denitrification. Fermentation to acetate or alanine is likely also a possibility, maintaining growth of these abundant lineages under anoxic conditions. Despite the relatedness of *Ca*. P. hodrii and *Ca*. P. baldrii, which are identical at the 16S rRNA gene V1-V3 ASV level, they coexist often both in high abundances in the same WWTPs, perhaps due to the differences in substrate use indicated by annotation of their genomes. Future experimental work will be needed to verify our hypothesis that these two abundant species find unique niches in WWTPs, possibly through the use of distinct and/or dynamically changing nutrient sources. Importantly, through FISH, Raman and phosphate cycling experiments, we confirm that these two species are both PAOs and store polyphosphate at amounts relevant to P recovery efforts. Collectively our data suggest that this PAO group is as important globally in WWTPs as *Ca*. Accumulibacter, if not in biovolume, then definitely in abundance and widespread distribution.

Future studies are required to comprehensively investigate the physiology of this important group in situ. *Ca*. Phosphoribacter species are so abundant in EBPR plants that previous in situ studies using FISH probes with broader coverage likely targeted them. FISH-Microautoradiography and phosphate cycling experiments showed capability for uptake of acetate, glucose, amino acids and possibly oleic acids [13, 70], but further experiments are now required to confirm this for *Ca*. Phosphoribacter and *Ca*. Lutibacillus. Their physiology is different from the two other dominant PAOs worldwide, i.e., *Ca*. Accumulibacter [5, 9, 71] and members of *Dechloromonas* [50], particularly regarding storage compounds, as they lack PHA and glycogen. In addition, the lack of full denitrification, the broad substrate uptake profile including glucose, and their fermenting capabilities indicate very different ecological niches. Interestingly, none of these PAOs have been isolated so far, suggesting some unknown growth requirements. Enrichment cultures have proven very useful for the investigation of *Ca*. Accumulibacter and should also be used to study *Dechloromonas, Ca*. Phosphoribacter and *Ca*. Lutibacillus spp. Importantly, this would enable us to uncover the energy storage compounds potentially used by *Ca*. Phosphoribacter and *Ca*. Lutibacillus, perhaps cyanophycin or free amino acids, to drive phosphate uptake and accumulation.

### Etymology of the newly redefined *Tetrasphaera*

Proposed etymologies and protologues for *Ca*. Phosphoribacter baldrii, *Ca*. Phosphoribacter hodrii and *Ca*. Lutibacillus vidarii are provided in Supplementary Tables 3, 4 and 5, respectively.

## Supplementary information

Supplementary material

Dataset1

Dataset2

Dataset3

Dataset4

Dataset5

Dataset6

Dataset7

Dataset8

Dataset9

## Data Availability

All data is available in NCBI under BioProject PRJNA622675 [18] for Danish MiDAS3 data, PRJNA629478 [19] for MiDAS genome database MAG and metagenome data, and PRJNA728873 [20] for global MiDAS4 data.
