# Supplementary material for: The novel genus, ‘Candidatus Phosphoribacter’, previously identified as Tetrasphaera, is the dominant polyphosphate accumulating lineage in EBPR wastewater treatment plants worldwide
Source: ISME J. 2022 Feb 25;16(6):1605–16. doi: 10.1038/s41396-022-01212-z (PMC9123174; doi:10.1038/s41396-022-01212-z)
Supplement: Supplementary file 1 — Supplementary material [file 41396_2022_1212_MOESM1_ESM.pdf]

Supplementary Information

The novel genus, '*Candidatus* Phosphoribacter', previously identified as *Tetrasphaera*, is the dominant polyphosphate accumulating lineage in EBPR wastewater treatment plants worldwide.

C.M. Singleton[1], F. Petriglieri[1], K. Wasmund[1,2], M. Nierychlo[1], Z. Kondrotaite[1], J.F. Petersen[1], M. Peces[1], M.S. Dueholm[1], M. Wagner[1,2], P.H. Nielsen[1*]

[1]Center for Microbial Communities, Department of Chemistry and Bioscience, Aalborg University, Aalborg, Denmark.

[2]Division of Microbial Ecology, Centre for Microbiology and Environmental Systems Science, University of Vienna, Vienna, Austria.

*Corresponding author: Prof. Per Halkjær Nielsen, Center for Microbial Communities, Department of Chemistry and Bioscience, Aalborg University, Fredrik Bajers Vej 7H, 9220 Aalborg, Denmark; Phone: (+45) 9940 8503; Email: phn@bio.aau.dk

Supplementary Figures

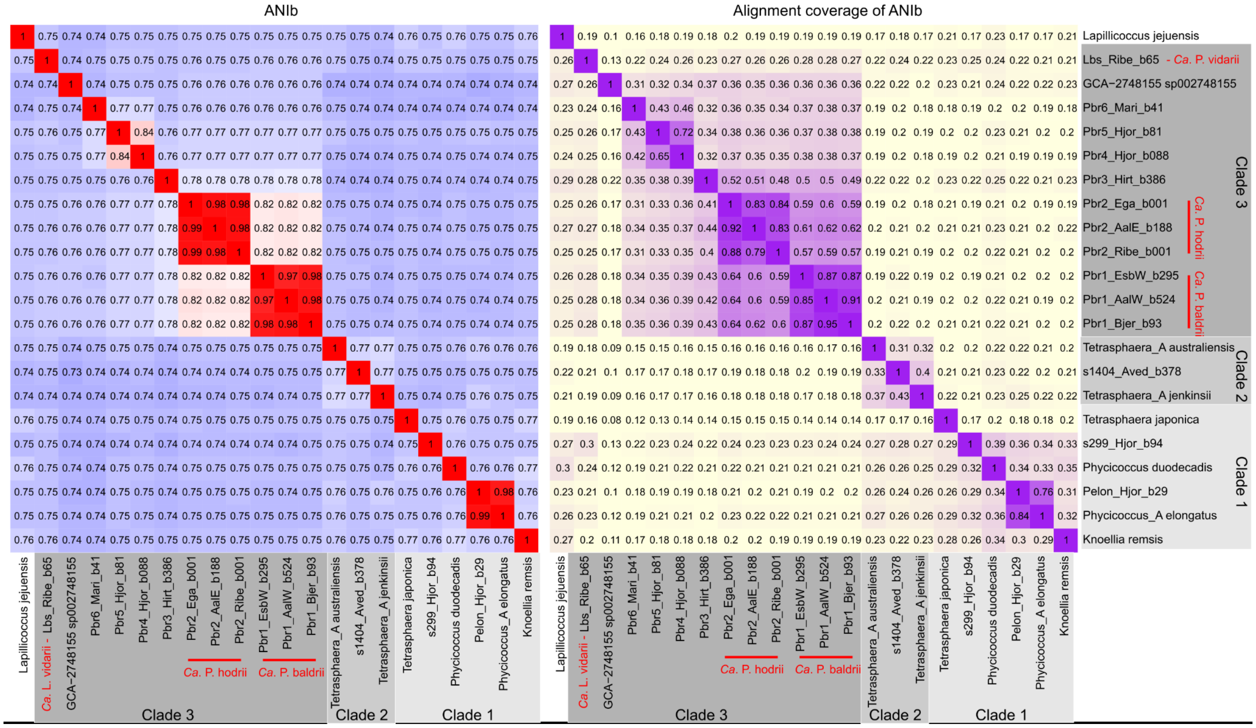

Supplementary Figure 1. ANIb of the MAGs and genomes belonging to the former *Tetrasphaera* and the proportion of genomes aligned (coverage).

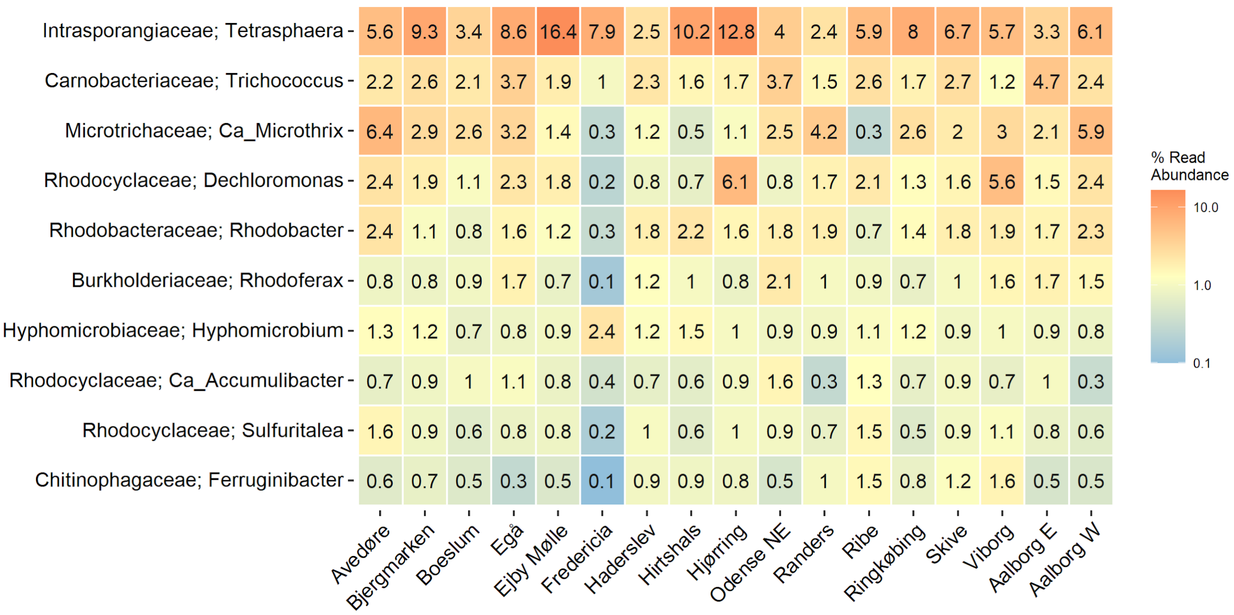

Supplementary Figure 2. Occurrence of the top 10 most abundant bacterial genera in Danish WWTPs with nitrogen and phosphorus removal. Family and genus names are shown. Data represents 612 samples from 17 EBPR plants and comes from the long-term (2006-2018) amplicon survey of microbial communities in Danish WWTPs [1].

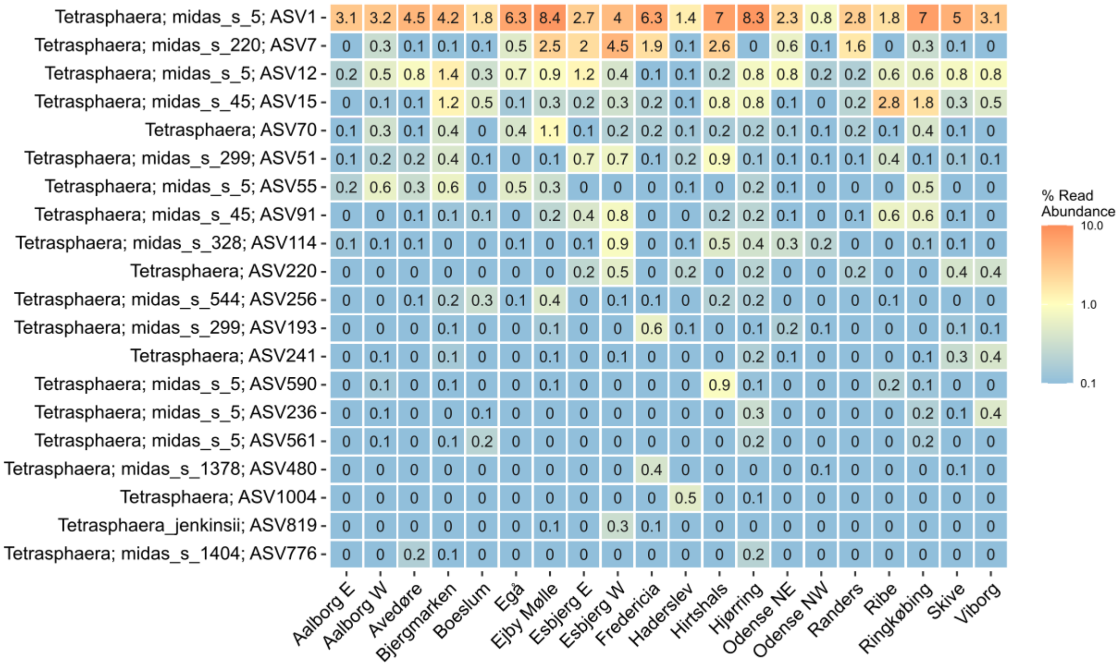

Supplementary Figure 3. The percent relative abundance of *Tetrasphaera* ASVs across Danish WWTPs in MiDAS3. Data represents 712 samples from 20 Danish nutrient removal WWTPs from the period 2006-2018 [1].

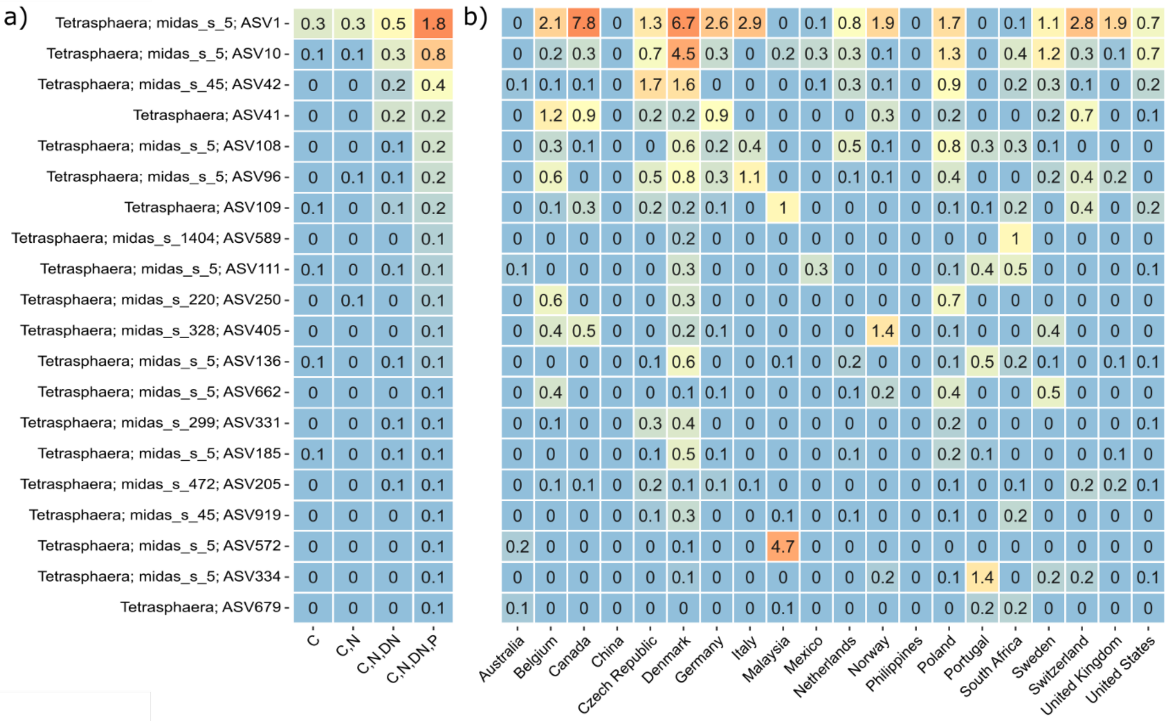

Supplementary Figure 4. Average percent relative read abundance of top 20 *Tetrasphaera* ASVs across a) different process configurations (N plants = 480; C - carbon removal; C,N - carbon removal and nitrification; C,N,DN - carbon and nitrogen removal; C,N, DN - carbon, nitrogen and phosphorus removal), (N plants = 480) and b) across the world in EBPR (C,N,DN,P) plants (N plants = 111). Taxa without species classification are shown only with their ASV number. Data comes from the global survey of microbial communities in WWTPs [2].

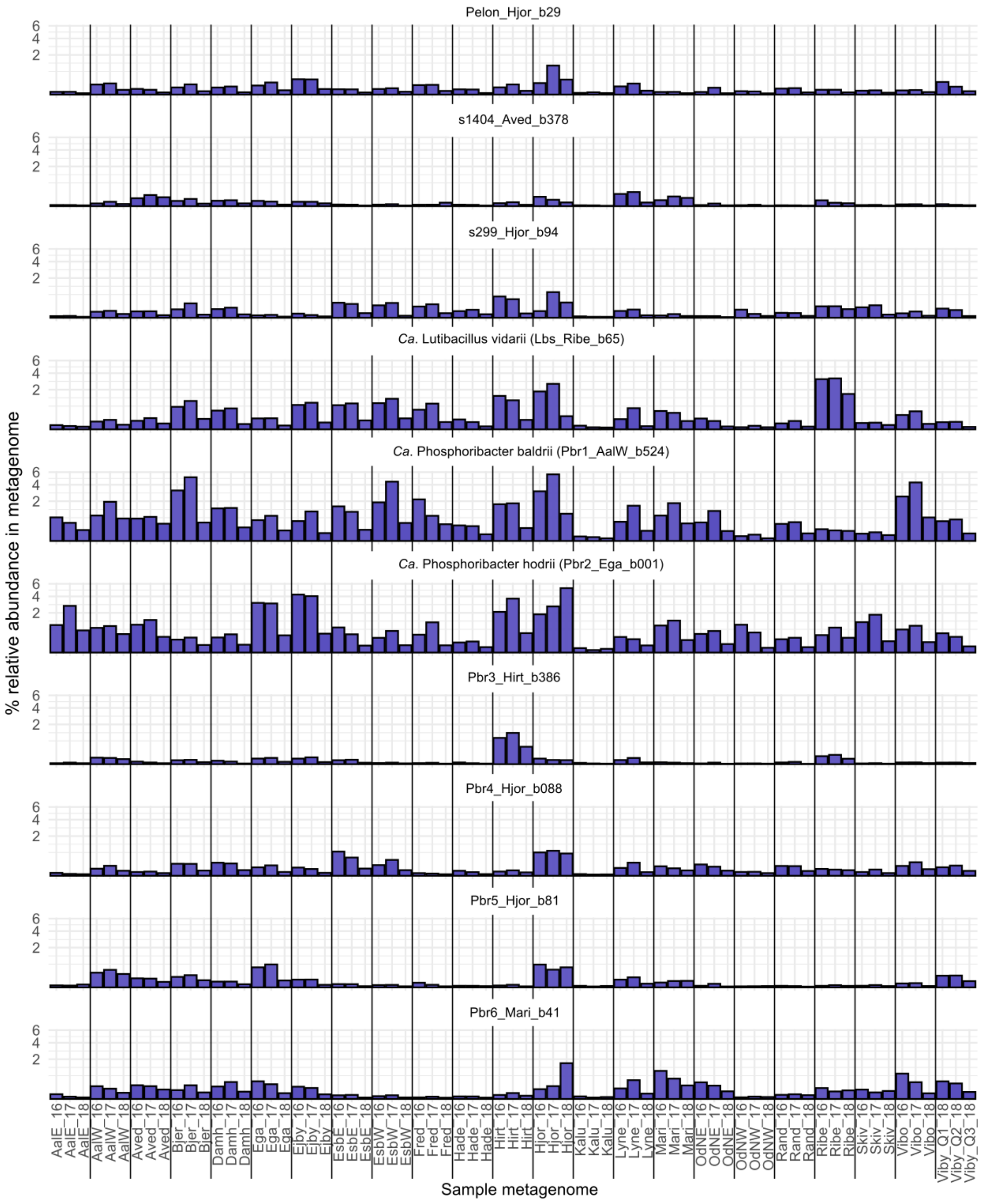

Supplementary Figure 5. Percent relative abundance of the 10 distinct former *Tetrasphaera* species across 69 Danish WWTP AS metagenomes [3], including resolution of the two midas_s_5 ASV1 species (*Ca*. P. baldrii and *Ca*. P. hodrii, represented by Pbr1_AalW_b524 and Pbr2_Ega_b001). Relative abundances were determined using stringent mapping of the reads (95% identity and 75% alignment) as in [3]. Metagenomes are shown across the x axis, lines indicate changes to different WWTPs, and three time points are shown for each WWTP. The percentage relative abundance from the reads that mapped compared to the metagenome is shown on the y axis. The axis is square root transformed. MAG IDs follow the naming in **Figure 1**.

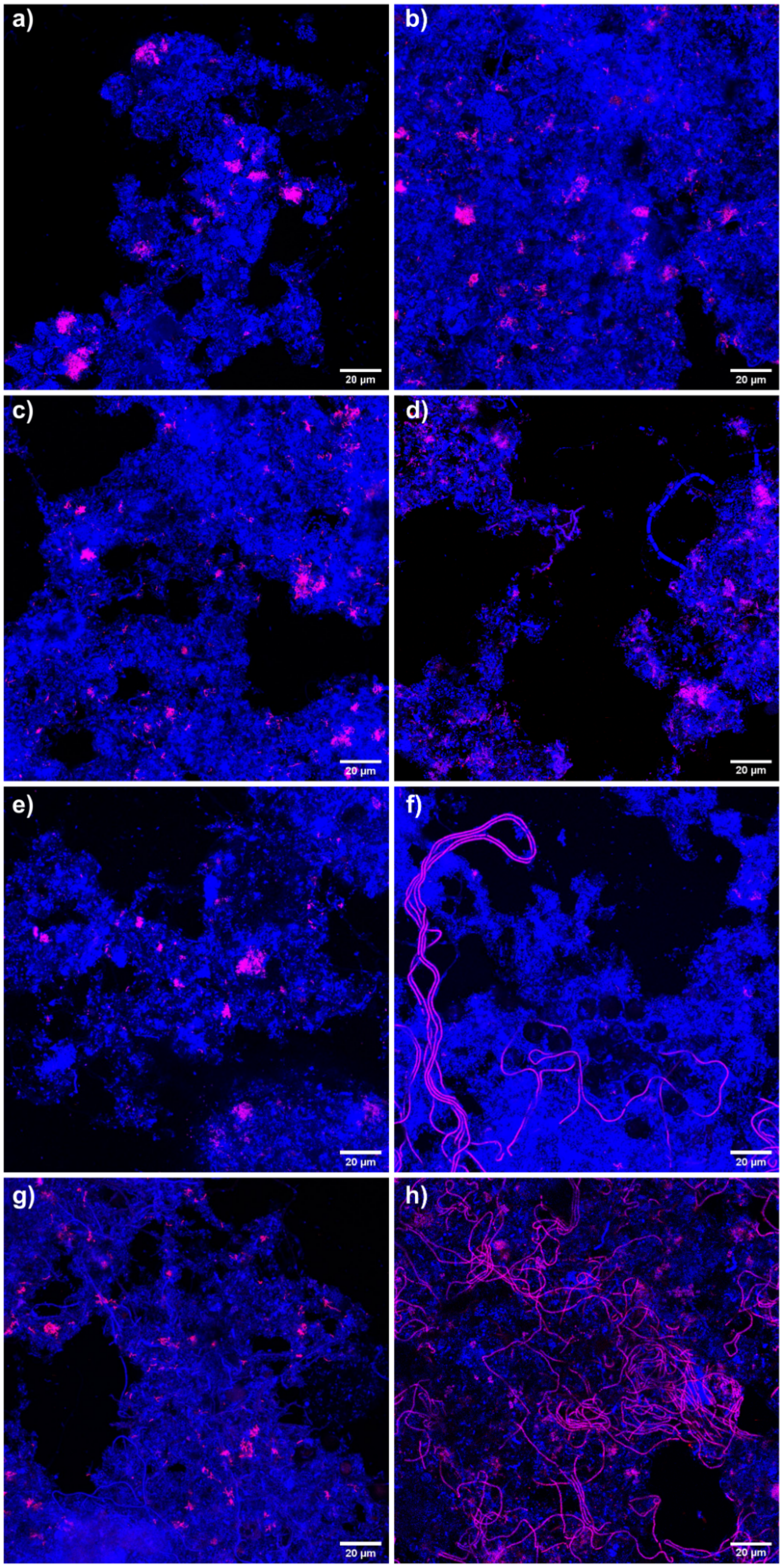
a)
20 µm
b)
20 µm
c)
20 µm
d)
20 µm
e)
20 µm
f)
20 µm
g)
20 µm
h)
20 µm

Supplementary Figure 6. Panel image of FISH micrographs. In all pictures all bacteria in blue are targeted by EUBmix, while bacteria of interest are targeted by specific probes in purple. **A)** *Ca.* Phosphoribacter hodrii visualised using the 16S rRNA probe (probe Phos601) (Horsens 2009 February)**. B)** *Ca.* P. hodrii visualised using the 23S rRNA probe (Phos1260-23S-Pbr2) (Horsens 2009 February). **C)** *Ca.* P. baldrii (Phos1260-23S-Pbr1) (Horsens 2009 February). **D)** All *Ca.* Phosphoribacter (mix of probes Phos741 and Actino658) (Horsens 2009 February). **E)** *Ca***.** Lutobacillus (Luti617) (Olszyn 2018 March). **F)** midas_s_328 (filamentous morphology) and midas_s_299 (Tetra732), no other target organisms were present in the sample according to amplicon sequencing (Aars 2010 February). **G)** midas_s_299 (Tetra67), no other target organisms were present in the sample according to amplicon sequencing (Aars 2010 February). **H)** Most of the former *Tetrasphaera*-related genera targeted by probe Tetra183 (Horsens 2009 February).

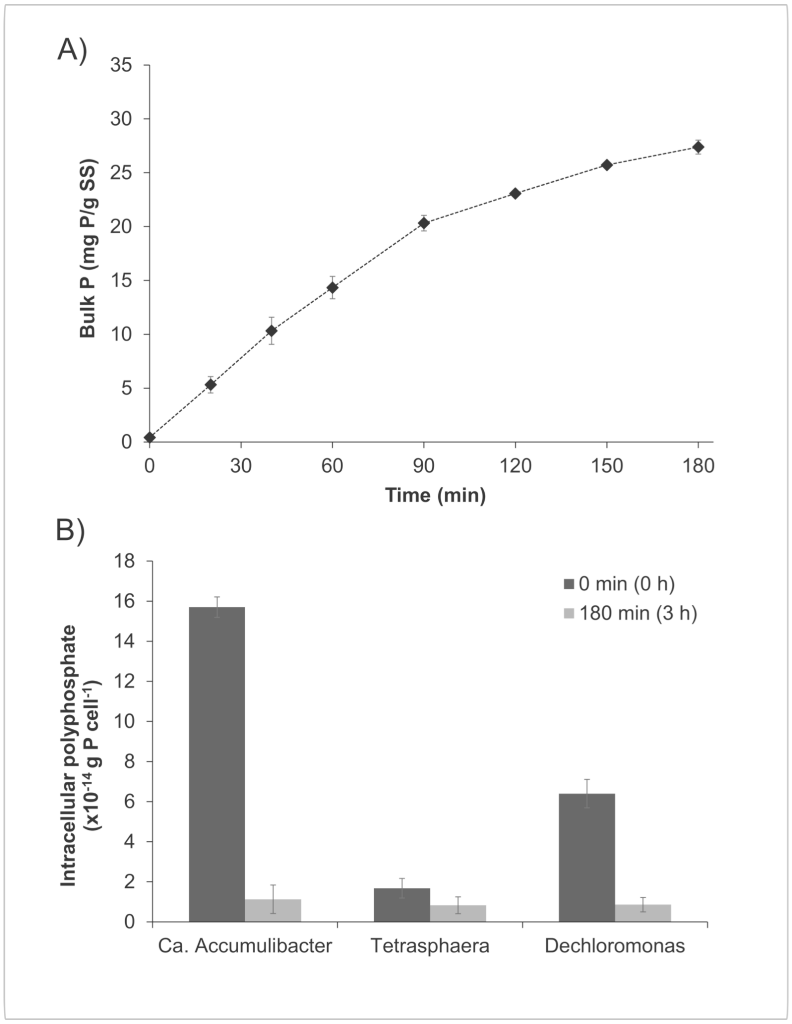

Supplementary Figure 7. P-release profile and polyP dynamics in cells of "Tetrasphaera", *Ca.* Accumulibacter and *Dechloromonas,* partly based on experiments presented elsewhere from the same WWTP and the same sample [4]. **A**) Bulk ortho-P concentration during the anaerobic P-release experiments with activated sludge from the Aalborg West WWTP. **B**) Total intracellular polyphosphate measured as an average of 1,500 randomly selected microbial cells by Raman microspectroscopy in initial oxic samples (0 h) and after anaerobic P-release (3 h). FISH probes covered all *Ca.* Accumulibacter (FISH probe PAO651), most "Tetrasphaera" (FISH probe Actino658), including *Ca.* Phosphoribacter hodrii and *Ca.* Phosphoribacter baldrii (see **Figure 5**), and *Dechloromonas* (FISH probe Bet135). Mass-balances based on cell count and polyP content showed that "Tetrasphaera" constituted approx. 22% of the total polyP, while *Ca.* Accumulibacter and *Dechloromonas* constituted approx. 14% and 6%, respectively [4].

Supplementary Tables

Supplementary Table 1. Comparative genomics of the *Ca.* P. baldrii (Pbr1, 3 MAGs) and *Ca.* P. hodrii (Pbr2, 3 MAGs) MAGs with 50% amino acid identity clustering at 80% alignment coverage. Output was collected from Microbial Genome Analysis and Annotation Platform, Magnifying Genomes (MaGe).

**Group**	**Component**	**Families**	**Genes**	**Excluding**
Ca. P. baldrii + *Ca.* P. hodrii	Pan-genome	6915	21499	NA
Ca. P. baldrii + *Ca.* P. hodrii	Core-genome	1594	11393	NA
Ca. P. baldrii + *Ca.* P. hodrii	Variable-genome	5321	10106	NA
Ca. P. baldrii	Pan-genome	4439	10714	NA
Ca. P. baldrii	Core-genome	2318	8016	NA
Ca. P. baldrii	Variable-genome	2121	2698	NA
Ca. P. baldrii	Pan-genome	2150	3230	excluding pan-genome of Pbr2
Ca. P. baldrii	Core-genome	344	1082	excluding pan-genome of Pbr2
Ca. P. baldrii	Variable-genome	1806	2148	excluding pan-genome of Pbr2
Ca. P. hodrii	Pan-genome	4766	10786	NA
Ca. P. hodrii	Core-genome	2060	7172	NA
Ca. P. hodrii	Variable-genome	2706	3614	NA
Ca. P. hodrii	Pan-genome	2476	3596	excluding pan-genome of Pbr1
Ca. P. hodrii	Core-genome	320	1018	excluding pan-genome of Pbr1
Ca. P. hodrii	Variable-genome	2156	2578	excluding pan-genome of Pbr1

Supplementary Table 2. Probes used in this study. * Taxonomy and coverage of groups is defined as an in the MiDAS 4.8 database [2]. Values given as group hits/ group totals; ** Recommended optimal formamide concentration for use in FISH hybridisations; N/A – not applicable. # The probes hybridise to the 23S rRNA of the target organisms.

Probe	*E. coli* pos.	Target group	Target molecule	Coverage*	Non-target hits	Sequence (5'-3')	[FA]%**	Reference
**Actino221**	**221-238**	**midas_s_220**	16S rRNA	**6/6**	**0**	**CGC AGG TCC ATC CCA GAC**	**30**	**Kong et al. 2005**
Actino221_C1	221-238	Competitor for Actino221	16S rRNA	N/A	N/A	CGC AGG TCC ATC CCA TAC	N/A	Kong et al. 2005
Actino221_C2	221-238	Competitor for Actino221	16S rRNA	N/A	N/A	CGC GAG TCC ATC CCA GAC	N/A	Kong et al. 2005
Actino221_C3	221-238	Competitor for Actino221	16S rRNA	N/A	N/A	CGC AGG TCC ATC CCA GAG	N/A	16S rRNA
**Actino658**	**658-675**	***Ca.* Phosphoribacter (*Ca.* P. baldrii, *Ca.* P. hodrii, Pbr3)**	16S rRNA	**24/67**	**0**	**TCC GGT CTC CCC TAC CAT**	**40**	**Kong et al. 2005**
Actino658_C1	658-675	Competitor for Actino658	16S rRNA	N/A	N/A	TCC GGT CTC CCC TAC CAC	N/A	Kong et al. 2005
Actino658_C2	658-675	Competitor for Actino658	16S rRNA	N/A	N/A	ATT CCA GTC TCC CCT ACC AT	N/A	Kong et al. 2005
Actino658_C3	658-675	Competitor for Actino658	16S rRNA	N/A	N/A	ATT CCA GTC TCC CCT ACC AC	N/A	This study
**Phos741**	**741-763**	***Ca.* Phosphoribacter (Pbr4, Pbr5, Pbr6)**	16S rRNA	**34/67**	**0**	**TTC TCA GCG TCA GTT GTG GCC C**	**30**	This study
Phos741_C1	741-763	Competitor for Phos741	16S rRNA	N/A	N/A	TCC TCA GCG TCA GTT GTG GCC C	N/A	This study
Phos741_C2	741-763	Competitor for Phos741	16S rRNA	N/A	N/A	GTC TCA GCG TCA GTT GTG GCC C	N/A	This study
Phos741_C3	741-763	Competitor for Phos741	16S rRNA	N/A	N/A	TTC TCA GCG TCA GTT ATG GCC C	N/A	This study
Phos741_C4	741-763	Competitor for Phos741	16S rRNA	N/A	N/A	TTC TCA GCG TCA GTG GCC C	N/A	This study
**Phos601**	**601-625**	**midas_s_5 (*Ca.* P. hodrii)**	16S rRNA	**7/67**	**0**	**GGT TGA GCC TCG GAT TTT CAC TGC**	**30**	**This study**
Phos601_C1	601-625	Competitor for Phos601	16S rRNA	N/A	N/A	GGT TGA GCC CCG GAT TTT CAC TGC	N/A	This study
Phos601_C2	601-625	Competitor for Phos601	16S rRNA	N/A	N/A	GGT TGA GCC TCG GAT TTT CAC RGC	N/A	This study
**Phos1260-23S-Pbr1#**		***Ca.* Phosphoribacter baldrii**	23S rRNA			**AGA GTT CAC GGC CGG GCA AAG**	**30**	**This study**
**Phos1260-23S-Pbr2#**		***Ca.* Phosphoribacter hodrii**	23S rRNA			**AGA CAT CAC GGC CGG GCA ATG**	**35**	**This study**
**Luti617**	**617-641**	***Ca.* Lutibacillus**	16S rRNA	**5/13**	**0**	**CCC ACT GCA AGT CCG GAA TTG AGT**	**30**	**This study**
**Tetra732**	**732-751**	**midas_s_299, midas_s_328, midas_s_1378**	16S rRNA	**7/19, 3/3, 3/5**	**0**	**AGT GGT GGC CCA GAG ACC TG**	**40**	**This study**
Tetrra732_C1	732-751	Competitor for Tetra732	16S rRNA	N/A	N/A	AGT WGT GGC CCA GAG ACC TG	N/A	This study
**Tetra67**	**67-88**	**midas_s_299, midas_s_469, *T. elongata*, midas_s_24955, midas_s_24809, midas_s_35051, midas_s_5540, midas_s_31199**	16S rRNA	**9/19, 5/8, 12/26, 1/1, 1/1, 1/1, 2/2, 2/2**	**8**	**AGC AAG CTC CGT CAC CG**	**40**	**This study**
Tetra67_C1	67-88	Competitor for Tetra67	16S rRNA	N/A	N/A	AGC AAG CTC CWT CAC CG	N/A	This study
**Tetra183**		**Targets most of Tetrasphaera - related genera**	16S rRNA			**TAGAGATGCCTCTCCGTCTC**	**30**	**This study**
Tetra183_H1		Helper probe for Tetra183	16S rRNA	N/A	N/A	YCCAGAGTCTGGGGCAGGTT	N/A	This study
Tetra183_H2		Helper probe for Tetra183	16S rRNA	N/A	N/A	GTCCATCCCAGACCGAAAAACTTT	N/A	This study

Supplementary Table 3: Protologues for *Candidatus* Phosphoribacter baldrii

Species name	*Candidatus* Phosphoribacter baldrii
Genus name	*Candidatus* Phosphoribacter
Specific epithet	baldrii
Type species of the genus	*Candidatus* Phosphoribacter baldrii
Genus status	Candidatus
Species etymology	Phos.pho.ri.bac'ter. N.L. masc. n. *phosphorus* phosphorus; N.L. masc. n. *bacter* rod; N.L. masc. n. *Phosphoribacter* a rod shaped bacterium that stores phosphate; bal.dri.i. N.L. gen. n. *baldrii* of Baldr, Norse god twin of Hodr, indicating the close relationship of the 16S rRNA gene sequence for *Phosphoribacter baldrii* and *Phosphoribacter hodrii*
Species status	sp. nov.
Designation of the type MAG	GCA_016704565.1
MAG/SAG accession number	GCA_016704565.1
Genome status	High-quality draft
Genome size	3605160
GC mol %	69.3
Country of origin	Denmark
Region of origin	Aalborg
Source of sample	Full-scale biological nutrient removal wastewater treatment plant
Geographical location	Aalborg
Latitude	57.049513 N
Longitude	9.864788 E
Depth	N/A
Altitude	N/A
Temperature of the sample	Mesophilic
pH of the sample	N/A
Relationship to oxygen	facultative anaerobe
Energy metabolism	Likely utilises a range of substrates including sugars and amino acids, is capable of polyphosphate accumulation and cycling, with the distinction of potential ethanolamine use
Assembly	1 sample
Sequencing technology	Oxford Nanopore and Illumina Hiseq X
Binning software used	MetaBAT2
Assembly software used	CANU v1.8
Habitat	Full-scale biological nutrient removal wastewater treatment plant
Miscellaneous, extraordinary features relevant for the description	Rod shaped cells 0.4-0.7 x 1-1.7 µm (diameter x length), usually organized in microcolonies embedded in the structure of the floc

Supplementary Table 4: Protologues for *Candidatus* Phosphoribacter hodrii

Species name	*Candidatus* Phosphoribacter hodrii
Genus name	*Candidatus* Phosphoribacter
Specific epithet	hodrii
Type species of the genus	*Candidatus* Phosphoribacter baldrii
Genus status	Candidatus
Species etymology	Phos.pho.ri.bac'ter. N.L. masc. n. *phosphorus* phosphorus; N.L. masc. n. *bacter* rod; N.L. masc. n. *Phosphoribacter* a rod shaped bacterium that stores phosphate; ho'dri.i. N.L. gen. n. *hodrii* of Hodr, Norse god twin of Baldr, indicating the close relationship of the 16S rRNA gene sequence for *Phosphoribacter hodrii* and *Phosphoribacter baldrii*
Species status	sp. nov.
Designation of the type MAG	GCA_016707175.1
MAG/SAG accession number	GCA_016707175.1
Genome status	High-quality draft
Genome size	3,909,549
GC mol %	69.5
Country of origin	Denmark
Region of origin	Ega
Source of sample	Full-scale biological nutrient removal wastewater treatment plant
Geographical location	Ega
Latitude	56.21314 N
Longitude	10.242467 E
Depth	N/A
Altitude	N/A
Temperature of the sample	Mesophilic
pH of the sample	N/A
Relationship to oxygen	facultative anaerobe
Energy metabolism	Likely utilises a range of substrates including sugars and amino acids, is capable of polyphosphate accumulation and cycling, with the distinction of potential fermentation to acetate and N-acetylglucosamine use.
Assembly	1 sample
Sequencing technology	Oxford Nanopore and Illumina Hiseq X
Binning software used	Maxbin2
Assembly software used	CANU v1.8
Habitat	Full-scale biological nutrient removal wastewater treatment plant
Miscellaneous, extraordinary features relevant for the description	Rod shaped cells 0.4-0.7 x 1-1.7 µm (diameter x length), usually organized in microcolonies embedded in the structure of the floc

Supplementary Table 5: Protologues for *Candidatus* Lutibacillus vidarii

Taxonumber	N/A
Species name	*Candidatus* Lutibacillus vidarii
Genus name	*Candidatus* Lutibacillus
Specific epithet	vidarii
Type species of the genus	*Candidatus* Lutibacillus vidarii
Taxonumber of the type species	N/A
Genus status	Candidatus
Species etymology	Lu.ti.ba.cil'lus. L. neut. n. *lutum* mud, dirt; L. masc. n. *bacillus* small rod; N.L. masc. n. *Lutibacillus* a rod-shaped bacterium from sludge; vi.da'ri.i. N.L. gen. n. *vidarii* of Vidar, second strongest son of Norse god Odin, indicating the second most abundant polyphosphate accumulating genus after *Phosphoribacter*
Species status	sp. nov.
Designation of the type MAG	GCA_016717165.1
MAG/SAG accession number	GCA_016717165.1
Genome status	High-quality draft
Genome size	3,509,479
GC mol %	70.9
Country of origin	Denmark
Region of origin	Ribe
Source of sample	Full-scale biological nutrient removal wastewater treatment plant
Geographical location	Ribe
Latitude	55.329053 N
Longitude	8.74336 E
Depth	N/A
Altitude	N/A
Temperature of the sample	Mesophilic
pH of the sample	N/A
Relationship to oxygen	facultative anaerobe
Energy metabolism	Likely utilises a range of substrates including sugars and amino acids, and is capable of polyphosphate accumulation.
Assembly	1 sample
Sequencing technology	Oxford Nanopore and Illumina Hiseq X
Binning software used	MetaBAT2
Assembly software used	CANU v1.8
Habitat	Full-scale biological nutrient removal wastewater treatment plant
Miscellaneous, extraordinary features relevant for the description	Cocci (0.6-0.8) or rod-shaped cells (0.5-0.9 x 2-4 μm) (diameter x length), occasionally found in tetrads

Supplementary Data Files

Supplementary Data File 1: MAG statistics for former *Tetrasphaera* MAGs in the study

Supplementary Data File 2: 16S rRNA gene ANIb table for the 14 MAGs

Supplementary Data File 3: Accession numbers for the genomes included in the tree and CheckM results

Supplementary Data File 4: KO gene list for genome metabolisms shown in Figure 3 and 4

Supplementary Data File 5: Full KO gene hit list table from all *Dermatophilaceae* genomes analysed.

Supplementary Data File 6: KO module paths for KEGG modules that were found to be at least 80% complete

Supplementary Data File 7: EnrichM completeness of KO module paths for KEGG modules that were found to be at least 80% complete

Supplementary Data File 8: Ortho-group analysis of proteins from *Ca*. P. baldrii and *Ca.* P. hodrii.

Supplementary Data File 9: Overview of predicted extracellular peptidases identified among novel MAGs recovered from Danish WWTPs

Supplementary Methods

Fluorescence in situ hybridisation (FISH)

An optimal formamide concentration was determined for each novel FISH probe after carrying out hybridisation at different formamide concentrations (0-70% with increments of 5%). Where available, suitable pure cultures having defined mismatches in the rRNA probe target region were obtained from DSMZ and applied in the optimization process. *Sanguibacter suarezii* (DSM10543), *Lactobacillus reuteri* (DSM20016) and *Janibacter melonis* (DSM16063) were used to assess the need of the specific unlabelled competitor probes Tetra67_C1, Actino221_C3 and Tetra732_C1, respectively. If suitable pure cultures were not available, hybridisation conditions for probes were optimised by selecting activated sludge biomass with a high abundance of the target organism predicted by amplicon sequencing. Microscopic analysis was performed with Axioskop epifluorescence microscope (Carl Zeiss, Germany) equipped with LEICA DFC7000 T CCD camera or with white light laser confocal microscope (Leica TCS SP8 X). The intensity of at least 50 cells at each formamide concentration was measured with ImageJ [5]. Optimal hybridisation conditions and details on the coverage and specificity of the FISH probes can be found in **Supplementary Table 2**. The EUBmix probes [6, 7] and the NON-EUB probe [8] were used to target all bacteria and as a negative control for sequence independent probe binding, respectively. For multicolor FISH, 30% formamide concentration was selected to obtain optimal signal intensity, as it was

experimentally determined to be the optimal for all the probes used in the experiment, except one (Actino658). To avoid nonspecific binding of the latter, a sample with no organisms with 16S rRNA genes with less than three mismatches to the probe was selected.

Raman microspectroscopy

FISH was conducted on optically polished CaF_2 Raman windows (Crystran, UK). Cells with probe conferred fluorescence were located with a 50× dry objective (Olympus M Plan Achromat- Japan) of the in-built Olympus (model BX-41) fluorescence microscope. After bleaching of fluorophore-derived Raman signals, Raman spectra from single-cells were obtained using a Horiba LabRam HR 800 Evolution (Jobin Yvon – France) equipped with a Torus MPC 3000 (UK) 532 nm 341 mW solid-state semiconductor laser. The specific settings for the spectrophotometer were: 5% neutral density (ND) filters, 600 mm/groove diffraction grating, 100 μm and 72 μm slidth width and confocal pinhole, respectively. Raman spectra collected spanned the wavenumber region of 200 cm^{-1} to 3000 cm^{-1}. The Raman spectrometer was calibrated prior to obtaining all measurements to the first-order Raman signal of Silicon, occurring at 520.7 cm^{-1}. Raman spectrometer operation and subsequent processing of spectra were conducted using LabSpec version 6.4 software (Horiba Scientific, France). Absolute quantification of intracellular poly-P was carried out as described previously [9]. The method assumes that the intensity of the Raman signal is directly dependent on the amount of the analyte in a determined area. An average amount of poly-P per cell was calculated as a factor of a constant determined during calibration for poly-P, the average charge-coupled device (CCD) counts determined during the experiment, and the average area of cells measured by image analysis [9].

Supplementary Notes

Supplementary Note 1 - Comparative genomics extended discussion

Carbon sources, processing and adaptations to anaerobic growth

Transporters predicted for xylose, ribose and glucose were not widely distributed, particularly in clade 1, 2 and 3 *Tetrasphaera* groups (**Supplementary Data Files 6 & 7**). *P. duodecadis* was an exception encoding a range of sugar transporters, and *T. australiensis* also has the potential for fructose and xylose import. However, three additional ABC sugar transporters, two putative and one multiple sugar transporter, are encoded in the former *Tetrasphaera* isolate genomes and MAGs (**Supplementary Data Files 6 & 7**). As previous isolates have shown growth on glucose [10], it is likely these transporters facilitate import of a range of simple sugars such as glucose and xylose [11]. Fructose is another potential carbon source for the *Ca*. Phosphoribacter group, which encoded a PTS sugar transporter subunit IIABC component (*fruB,* KO

number: K02768), a 1-phosphofructokinase (*fruK* K00882) and a fructose operon transcriptional repressor (*fruR* K03436) all adjacent to each other.

Glycerol 3-phosphate could also potentially be used as a carbon and phosphate source by Pbr3-6 based on the presence of an ABC transporter encoded by the *ugpABE* (K05814, K05813, K05815) and *malK* (K10112) genes, and processed via glycolysis (**Figure 3**) [12].

Use of lactate as a carbon source by the clade 3 group is similarly indicated by the presence of lactate utilisation proteins BCA (*lldG* K00782, *lldF* K18929, *lldE* K18928), lactate permease (*lctP* K03303), L-lactate dehydrogenase (*lldD* K00101) and a lactate-response regulator (*gntR* K05799). Potential for acetate transport (*actP* K14393) and the use of acetate as a carbon source through either *acs* (K01895) or *ackA* (K00925) and *pta* (K13788) was identified in most TRC genomes (**Figure 3**) [13]. Additionally, most TRC encoded the pyruvate dehydrogenase (*aceE* K00163, *aceF* K00627) for pyruvate oxidation to acetyl-CoA under oxic conditions.

The potential for beta-oxidation was also widely distributed across the TRC. While transporters for long-chain fatty acids (*fadL*) were missing, the acyl-CoA synthetase (K01897 *fadD*) specific for C6 to C18 fatty-acid biosynthesis or degradation was present in 68 of the 69 TRC genomes as were the genes for beta oxidation (K00249 *acd*, K01782 *fadJ*, K00632 *fadA*) [14]. However, the beta oxidation enzymes overlap with those involved in isoleucine, valine or leucine degradation, consequently it is difficult to determine whether long-chain fatty acids are degraded or only synthesised. The glyoxylate cycle was complete (with K01637 *aceA* isocitrate lyase) in only two genomes, *Terracoccus luteus* and the MAG GCA-2748155.

Fermentation of substrates to acetate, lactate, alanine and succinate has been determined in the former *Tetrasphaera* isolates either through experimental measurements or based on genomic potential [10]. The clade 3 *Ca*. Phosphoribacter (midas_s_5) MAGs also encode the pyruvate:ferredoxin oxidoreductase (*porA* K00169, *porB* K00170), which works to convert pyruvate to acetyl-CoA under anoxic or microoxic conditions [15, 16], indicating an adaptation to oxygen limited environments. All former clade 3 and nearly all TRC MAGs (58/69) encoded the alanine dehydrogenase (*ald* K00259) for the reduction of pyruvate to alanine. This action is reversible and potentially involved in maintaining redox balances and could facilitate anaerobic growth or alanine use similar to other Actinobacteriota [17]. However, only the *Ca*. P. hodrii and Pbr3 MAGs encoded the full fermentation to acetate pathway, which is missing in the other *Ca*. Phosphoribacter MAGs (missing *pta* and *ackA*) (**Figure 3**). Most TRC MAGs (61/69), including clade 3, encode the cytochrome *bd* oxidase (cydA K00425, cydB K00426), less efficient but better suited to low oxygen conditions than the cytochrome *c* oxidase [18], again indicating versatility suited to fluctuating oxygen conditions in EBPR systems.

Clade 3 MAGs were enriched for all three genes encoding standard formate dehydrogenase (*fdoGHI*, K00123, K00124, K00127) and an operon encoding many subunit of a putative formate-hydrogen lyase-like complex (*hycE* K15830, *hyfFECB* K12141, K12140, K12138, K12137, *mbhJ* K18023 and *arsR* K03892), indicating they have the capacity to dissipate formate that may accumulate from anaerobic fermentation, producing CO_2 and hydrogen [19]. Genes for indolepyruvate oxidoreductases (*iorA* K00179, *iorB* K00180) were also present in 8/10 clade 3 MAGs but were absent from nearly all of the remaining TRC (**Figure 3**). These typically oxygen sensitive enzymes may be used during anaerobic aromatic amino acid fermentation [20]. Overall, the clade 3 populations have various adaptations for anaerobic and/or microaerophilic growth compared to the former *Tetrasphaera* isolates, which indicates differences in their potential to utilise organics, and possibly fulfil unique nutrient niches in doing so.

Nitrogen cycling

Nitrogen cycling is an important target for optimisation and sustainability improvements in WWTP [21]. Consequently, we examined the distribution of nitrogen metabolism genes across the TRC. The potential for nitrate reduction to ammonia was prevalent in the *Knoellia*, *Janibacter*, *Pedococcus* and *Terrabacter* genera, but lacking in all former *Tetrasphaera* clades, except for *T. japonica*. A respiratory nitrate reductase (NarGHI, K00370, K00371, K00374) was encoded in all former *Tetrasphaera* isolates except *K. remsis* and *T. jenkinsii*. Similarly, most MAGs from the Danish WWTPs also encoded enzymes for nitrate reduction, with the exceptions being MAGs s1404_Aved_b378, Pbr1_EsbW_b295, Pbr3 and Pbr5. A capacity for nitrite reduction was more limited, with NirK (K00368) identified only in *Ca*. P. hodrii, Pbr3, Pbr4, Pbr5 of clade 3. *T. australiensis* of clade 2, and *P. duodecadis* and the MAG Pelon_Hjor_b29 (clade 1) also encoded a nitrite reductase. Using MAGE, manual inspection of the *T. japonica*, *P. elongatus* and *T. jenkinsii* revealed the presence of NirK homologues, which were missed in the KEGG orthology analysis. Nitric oxide reductase (NorBC K04561, K02305) was missing in all TRC genomes, and nitrous oxide reductase (NosZ K00376) was missing in all but one (UBA4719 sp002404345), showing the group is devoid of complete denitrifiers. Overall, the differences in nitrate and nitrite reduction potential across the group indicate some niche differentiation for respiration under anaerobic conditions using nitrate or nitrite as electron acceptors.

Polyphosphate accumulation

We examined the prevalence of genes important for, but not limited to, polyphosphate accumulation and storage. These genes were identified widely across the TRC (**Figure 3**). Nearly all MAGs encoded the low-affinity phosphate transporter Pit (K03306). The high affinity phosphate transporter encoded by PstSCAB (K02040, K02037, K02038, K02036) was also prevalent across the TRC, but less widespread than Pit, and missing in a few of the MAGs. One MAG in each of the *Ca*. P. baldrii and *Ca*. P. hodrii species missed the PstSCAB, but as two of the three MAGs in each species cluster encoded it, an absence could be due to

genome incompleteness or indicate strain variation. At the genomic level, polyphosphate accumulation appears possible for many members of the *Dermatophilaceae*, but the environmental conditions likely determine the storage and cycling phenotype, and as always experimental evidence is required for confirmation of this metabolic trait (see below).

Glycogen, PHA and amino acid storage

Additional storage compounds, such as glycogen and PHA, are believed to be integral to the PAO phenotype by providing energy for polyP accumulation during aerobic conditions [10]. None of the TRC genomes encoded all genes for PHA synthesis (PhaABC, K00626, K00023, K03821), with PhaA and PhaB or PhaA and PhaC found in only 15 of the 69 genomes (**Figure 3**). Two *Ca.* P. hodrii MAGs and *T. japonica* encoded PhaA and PhaC, and PHA has been detected using gas chromatography in *T. japonica [10]*, suggesting that *Ca.* P. hodrii may also be capable of PHA storage, however no PHA was detected experimentally (see below). Previous genome studies predicted *Tetrasphaera* produced glycogen as an energy storage compound [10], although recent work showed glycogen was not detectable in individual FISH-defined *Tetrasphaera* cells by Raman microspectroscopy in activated sludge samples from EBPR plants [9]. Genes for glycogen synthesis were identified in many TRC genomes (**Figure 3**), however we propose that the TRC may synthesize glycogen-like α-glucan polysaccharides as cell-wall capsular material, similar to other Gram-positive Actinobacteriota, rather than glycogen for storage.

Clustered among several genes previously assigned for glycogen synthesis in *P. elongatus* (i.e., *glgB*, *glgP*, *glgX*, *glgY*) [10], we identified several genes encoding enzymes for trehalose and maltose conversions, which together resemble the 'TreS-Pep2-GlgE' pathway for capsular glycogen synthesis in *Mycobacterium tuberculosis* [22, 23] (MetaCyc pathway 'glycogen biosynthesis III'). These gene complements were present in all novel MAGs and former *Tetrasphaera* isolates. Considering that the TRC are also Gram-positive Actinobacteriota, it is likely TRC bacteria also produce capsular polysaccharides using this pathway or a variation thereof.

P. elongatus is believed to be capable of accumulation of amino acids under anaerobic conditions, which are used as an energy source to take up phosphate during aerobic conditions [24]. We explored the presence of amino acid transporter genes in order to investigate if amino acids could be important energy sources for the clade 3 lineages, too. Genes encoding a transcriptional regulator and branched chain amino acid ABC transporters *livKHMGF* (K01995-K01999) were found across the TRC, and were present in multiple copies within the clade 3 MAGs, including up to five copies in *Ca*. P. hodrii (range 1-5) (**Figure 3**) [25]. Similarly, polar amino acids are likely transported via another detected putative ABC transporter (K02028-K02030) (**Figure 3**). This indicates that amino acids such as lysine, arginine, histidine, leucine, isoleucine, valine and phenylalanine are likely important growth substrates. Several key enzymes for aromatic amino acid catabolism and aromatic molecule catabolism were also enriched among the clade 3 MAGs, such as 1,2-

phenylacetyl-CoA epoxidase (*paaABCDE*, K02609-K02613) (phenylacetate, aerobic), phenylacetyl-CoA ligase (*paaK* K01912) (phenylacetate, anaerobic), gentisate 1,2-dioxygenase (K00450) (benzoates, cresols, aerobic [26]), and 2-ketocyclohexanecarboxyl-CoA hydrolase (*badI*, K07536) (benzoate) (**Figure 3**). Interestingly, while uncommon in bacteria, Actinobacteriota often encode and make use of proteasomes for degrading proteins [27], and 63/69 TRC genomes encoded them (**Supplementary Data Files 4 & 6**). Proteasomes could give the clade 3 lineages an advantage over *Ca*. Accumulibacter, enabling them to recycle resources from proteins and potentially respond quickly to challenging and fluctuating conditions [28], such as those in WWTPs.

Supplementary Note 2 - Difference between the most abundant species Ca. L. badrii and hodrii extended discussion

The metabolism unique to *Ca.* P. baldrii (and *Ca.* Lutibacillus vidarii in the TRC) is the potential use of ethanolamine using an ethanolamine utilisation operon *eutNABCLEMQJ* with an alcohol dehydrogenase and an *araC* (*eutR* - MAGE) family transcriptional regulator [29] (**Figure 4**). This operon is much longer than those previously detected in Actinobacteriota, which normally comprise only *eutBC* (K03735, K03736) and a transporter [30]. Ethanolamine is present in the membranes of all living cells as the lipid phosphatidylethanolamine, and would be readily available in the AS system, and able to diffuse across cell membranes at a neutral pH or via the EutH transporter [29]. Ethanolamine is a source of both carbon (acetaldehyde) and nitrogen (ammonia) and is likely processed in an organelle-like microcompartment that would contain the toxic and gaseous acetaldehyde [30]. Potentially, the acetaldehyde dehydrogenase (*eutE* K00132) can process acetaldehyde to acetyl-CoA, which can be used in the TCA cycle. The maintenance cost of such a complex operon and metabolism is high [29], suggesting that this pathway is used and could differentiate the *Ca*. P. baldrii niche from the similarly abundant *Ca*. P. hodrii.

Ca. P. hodrii encodes several metabolic pathways distinct from *Ca*. P. baldrii. These included the capacity for assimilatory sulfate reduction and siroheme biosynthesis (ssuBC K02049-K02050, cysN K00956, cysD K00957, cysH K00390, sir K00392, cysG K02302), as well as a long protoheme synthesis operon (*hemABCDEHL*, K02492, K01698, K01749, K01719, K01599, K01772, K01845). The potential to use the sugar N-acetylglucosamine as a carbon and nitrogen source, similar to *T. remsis* [31], is suggested by the presence of the N-acetylglucosamine-6-P deacetylase and deaminase (*nagA* K01443, *nagB* K02564)*,* PTS transporter genes (*nagE* K02802-K2804) and YvoA (K03710) regulator in all three MAGs. Use of this sugar is uncommon in WWTP microorganisms, indicating a distinct niche for this population [32]. This species also encoded the potential for aerobic acetate production from acetyl-CoA, acetate uptake, or fermentation of pyruvate to acetate via the *pta* (K13788) and *ackA* (K00925) genes, both of which were missing in the *Ca*. P. baldrii MAGs (**Figure 4**). Aerobic acetate production results from an overflow metabolism during exponential growth in *Escherichia coli* K12, which is hypothesised to be a consequence

of reaching metabolic capacity limits in the TCA cycle, respiratory chain, or acetyl-CoA concentrations [33]. Under anaerobic conditions the Pta and AckA can act in reverse to produce acetate and ATP from acetyl-CoA [13], thereby suggesting this population inhabits a different anaerobic niche to *Ca*. P. baldrii.

Supplementary Note 3 - FISH details

Genus- and, when possible, species-specific FISH probes (**Supplementary Table 2**) were designed to cover the most abundant species in each clade, showing a variety of different morphologies. The existing FISH probes Actino658 and Actino221 [34] target with high specificity and good coverage part of *Ca*. Phosphoribacter (*Ca*. P. baldrii, *Ca*. P. hodrii and Pbr3) and midas_s_220, respectively (**Supplementary Figure 6, Supplementary Table 2**). When applied in situ, Actino658 hybridized with rod-shaped cells (0.4-0.7 × 1-1.7 µm), usually organized in microcolonies embedded in the structure of the floc (**Supplementary Figure 6D**), while Actino221 targeted coccoid cells (0.8 - 0.9 µm) organized in tetrads. The FISH probe Phos741 was designed to cover the remaining part of *Ca*. Phosphoribacter (Pbr4, Pbr5, Pbr6) and hybridised to rod-shaped bacteria cells, with similar morphology as Actino658 (**Supplementary Figure 6D**). The FISH probe Phos601, which targets *Ca*. P. hodrii-related sequences (**Supplementary Figure 6A**), was the only 16S rRNA species-specific probe that was possible to design and optimise for this microorganism and its application confirmed the morphology already observed with Actino658. Two additional probes, Phos1260-23S-Pbr1 and Phos1260-23S-Pbr2, targeting 23S rRNA, were designed to specifically distinguish between *Ca*. P. baldrii and *Ca*. P. hodrii (**Supplementary Table 2**) and both hybridised with rod-shaped cells. Specificity of the species-specific probes was assessed by overlap with broader probes (**Supplementary Figure 6B-C**).

The FISH probe Luti617 was designed to target midas_s_45 and it showed rod-shaped (0.8-0.9 ×1.2-1.3 µm) or cocci-shaped (0.6-0.8) cells, occasionally found in tetrads (**Supplementary Figure 6E**). As it was not possible to design a specific probe for the species midas_s_299, the probes Tetra732 and Tetra67 were both designed to target it and their overlap is unequivocally identifying the species. However, these probes are also targeting low abundant species, midas_s_328 and midas_s_1436 for Tetra732 and *P. elongatus* and midas_s_15631 for Tetra67. They therefore hybridise with cells with different morphologies: Tetra732 is hybridising to rod-shaped cells (0.5-0.6 × 1.1-1.2 µm) and long filaments (1.2-1.3 × >100 µm), while Tetra67 is targeting the same rod-shaped cells (0.5-0.6 × 1.1-1.2 µm) and thinner filaments (0.6-0.7 × 20-90 µm) (**Supplementary Figure 6F-G**).

Application of the FISH probes with Raman microspectroscopy revealed the presence of polyP in all the genera/species (**Table 2**). No other storage polymers have been detected *in situ*, as previously observed [4, 9]. In order to quantify and explore the dynamics of polyP in the *Ca*. Phosphoribacter, we performed

anaerobic-aerobic P-cycling experiments with fresh activated sludge from a full-scale EBPR plant. Different carbon sources (acetate, glucose and casamino acids) were used during the anoxic phase as *Tetrasphaera* is known to use amino acids or sugars as substrates for P release under anoxic conditions [9, 35]. In situ quantification of polyP was performed for the two most abundant species, *Ca*. P. baldrii and *Ca*. P. hodrii. Both species exhibited dynamic cycling of intracellular polyP (**Figure 5C**), higher after the oxic phase and substantially decreasing after the anoxic phase, with small variations between the two species. The highest value was measured for *Ca*. P. hodrii ($1.82*10^{-14}$g P $cell^{-1}$), while *Ca*. P. baldrii accumulated $1.68*10^{-14}$ g P $cell^{-1}$ (**Figure 5B**). These values are in complete accordance with polyP contents measured with the same method but using the broader FISH probe Actino658 [4].

Etymology

Lu.ti.ba.cil'lus. L. neut. n. *lutum* mud, dirt; L. masc. n. *bacillus* small rod; N.L. masc. n. *Lutibacillus* a rod shaped bacterium from sludge; vi.da'ri.i. N.L. gen. n. *vidarii* of Vidar, second strongest son of Norse god Odin, indicating the second most abundant polyphosphate accumulating genus after *Phosphoribacter.*

Phos.pho.ri.bac'ter. N.L. masc. n. *phosphorus* phosphorus; N.L. masc. n. *bacter* rod; N.L. masc. n. *Phosphoribacter* a rod shaped bacterium that stores phosphate; bal.dri.i. N.L. gen. n. *baldrii* of Baldr, Norse god twin of Hodr, indicating the close relationship of the 16S rRNA gene sequence for *Phosphoribacter baldrii* and *Phosphoribacter hodrii.*

Phos.pho.ri.bac'ter. N.L. masc. n. *phosphorus* phosphorus; N.L. masc. n. *bacter* rod; N.L. masc. n. *Phosphoribacter* a rod shaped bacterium that stores phosphate; ho'dri.i. N.L. gen. n. *hodrii* of Hodr, Norse god twin of Baldr, indicating the close relationship of the 16S rRNA gene sequence for *Phosphoribacter hodrii* and *Phosphoribacter baldrii.*

References

1. Nierychlo M, Andersen KS, Xu Y, Green N, Jiang C, Albertsen M, et al. MiDAS 3: An ecosystem-specific reference database, taxonomy and knowledge platform for activated sludge and anaerobic digesters reveals species-level microbiome composition of activated sludge. Water Res. 2020;**182**:115955.
2. Dueholm MS, Nierychlo M, Andersen KS, Rudkjøbing V, Knudsen S, the MiDAS Global Consortium, et al. MiDAS 4: A global wastewater treatment plant ecosystem-specific full-length 16S rRNA gene catalogue and taxonomy for studies of bacterial communities across the world. BioRxiv. 2021. doi:https://doi.org/10.1101/2021.07.06.451231.
3. Singleton CM, Petriglieri F, Kristensen JM, Kirkegaard RH, Michaelsen TY, Andersen MH, et al. Connecting structure to function with the recovery of over 1000 high-quality metagenome-assembled genomes from activated sludge using long-read sequencing. Nat Commun. 2021;**12**:2009.
4. Petriglieri F, Petersen JF, Peces M, Nierychlo M, Hansen K, Baastrand CE, et al. Quantification of biologically and chemically bound phosphorus in activated sludge from full-scale plants with biological P-removal. BioRxiv. 2021. doi: https://doi.org/10.1101/2021.01.04.425262.
5. Schneider CA, Rasband WS, Eliceiri KW. NIH Image to ImageJ: 25 years of image analysis. Nat Methods. 2012;**9**:671–675.
6. Amann RI, Binder BJ, Olson RJ, Chisholm SW, Devereux R, Stahl DA. Combination of 16S rRNA-targeted oligonucleotide probes with flow cytometry for analyzing mixed microbial populations. Appl Environ Microbiol. 1990;**56**:1919–1925.
7. Daims H, Brühl A, Amann R, Schleifer KH, Wagner M. The domain-specific probe EUB338 is insufficient for the detection of all Bacteria:development and evaluation of a more comprehensive probe set. Syst Appl Microbiol. 1999;**22**:434–444.
8. Wallner G, Amann R, Beisker W. Optimizing fluorescent in situ hybridization with rRNA-targeted oligonucleotide probes for flow cytometric identification of microorganisms. Cytometry. 1993;**14**:136–143
9. Fernando EY, McIlroy SJ, Nierychlo M, Herbst FA, Petriglieri F, Schmid MC, et al. Resolving the individual contribution of key microbial populations to enhanced biological phosphorus removal with Raman–FISH. ISME J. 2019;**13**:1933–1946.
10. Kristiansen R, Nguyen HTT, Saunders AM, Nielsen JL, Wimmer R, Le VQ, et al. A metabolic model for members of the genus *Tetrasphaera* involved in enhanced biological phosphorus removal. ISME J. 2013;**7**:543–554.
11. Zhao J, Binns AN. Characterization of the mmsAB-araD1 (gguABC) genes of *Agrobacterium tumefaciens*. J Bacteriol. 2011;193:6586–6596.
12. Chandravanshi M, Gogoi P, Kanaujia SP. Computational characterization of TTHA0379:A potential

glycerophosphocholine binding protein of Ugp ATP-binding cassette transporter. Gene. 2016;**592**:260–268.

13. Enjalbert B, Millard P, Dinclaux M, Portais J-C, Létisse F. Acetate fluxes in *Escherichia coli* are determined by the thermodynamic control of the Pta-AckA pathway. Sci Rep. 2017;**7**:42135.
14. Jimenez-Diaz L, Caballero A, Segura A. Pathways for the degradation of fatty acids in bacteria. Aerobic utilization of hydrocarbons, oils and lipids. Handbook of Hydrocarbon and Lipid Microbiology, doi:10.1007/978-3-319-39782-5_42-1.
15. Ragsdale SW. Pyruvate ferredoxin oxidoreductase and its radical intermediate. Chem Rev. 2003;**103**:2333–2346.
16. Erb TJ. Carboxylases in natural and synthetic microbial pathways. Appl Environ Microbiol. 2011;**77**:8466–8477.
17. Feng Z, Cáceres NE, Sarath G, Barletta RG. *Mycobacterium smegmatis* L-alanine dehydrogenase (Ald) is required for proficient utilization of alanine as a sole nitrogen source and sustained anaerobic growth. J Bacteriol. 2002;**184**:5001–5010.
18. Morris RL, Schmidt TM. Shallow breathing:bacterial life at low O_2. Nat Rev Microbiol. 2013;**11**:205–212.
19. McDowall JS, Murphy BJ, Haumann M, Palmer T, Armstrong FA, Sargent F. Bacterial formate hydrogenlyase complex. Proc Natl Acad Sci USA. 2014;**111**:E3948–56.
20. Berger M, Brock NL, Liesegang H, Dogs M, Preuth I, Simon M, et al. Genetic analysis of the upper phenylacetate catabolic pathway in the production of tropodithietic acid by *Phaeobacter gallaeciensis*. Appl Environ Microbiol. 2012;**78**:3539–3551.
21. Jetten MSM, Horn SJ, van Loosdrecht MCM. Towards a more sustainable municipal wastewater treatment system. Water Sci Technol. 1997;**35**:171–180.
22. Kalscheuer R, Syson K, Veeraraghavan U, Weinrick B, Biermann KE, Liu Z, et al. Self-poisoning of *Mycobacterium tuberculosis* by targeting GlgE in an α-glucan pathway. Nat Chem Biol. 2010;**6**:376–384.
23. Chandra G, Chater KF, Bornemann S. Unexpected and widespread connections between bacterial glycogen and trehalose metabolism. Microbiology. 2011;**157**:1565–1572.
24. Nguyen HTT, Kristiansen R, Vestergaard M, Wimmer R, Nielsen PH. Intracellular accumulation of glycine in polyphosphate-accumulating organisms in activated sludge, a novel storage mechanism under dynamic anaerobic-aerobic conditions. Appl Environ Microbiol. 2015;**81**:4809–4818.
25. Hosie AH, Poole PS. Bacterial ABC transporters of amino acids. Res Microbiol. 2001;**152**:259–270.
26. Romero-Silva MJ, Mendez V, Agullo L, Seeger M. Genomic and functional analyses of the gentisate and protocatechuate ring-cleavage pathways and related 3-hydroxybenzoate and 4-hydroxybenzoate peripheral pathways in *Burkholderia xenovorans* LB400. PLoS One. 2013;**8**:e56038.
27. von Rosen T, Keller LM, Weber-Ban E. Survival in Hostile Conditions: Pupylation and the

Proteasome in Actinobacterial Stress Response Pathways. Front Mol Biosci. 2021;**8**:685757.

28. Müller AU, Weber-Ban E. The bacterial proteasome at the core of diverse degradation pathways. Front Mol Biosci. 2019;**6**:23.
29. Kaval KG, Garsin DA. Ethanolamine utilization in Bacteria. MBio. 2018;**9**.
30. Tsoy O, Ravcheev D, Mushegian A. Comparative genomics of ethanolamine utilization. J Bacteriol. 2009;**191**:7157–7164.
31. Osman S, Moissl C, Hosoya N, Briegel A, Mayilraj S, Satomi M, et al. *Tetrasphaera remsis* sp. nov., isolated from the Regenerative Enclosed Life Support Module Simulator (REMS) air system. Int J Syst Evol Microbiol. 2007;**57**:2749–2753.
32. Kragelund C, Levantesi C, Borger A, Thelen K, Eikelboom D, Tandoi V, et al. Identity, abundance and ecophysiology of filamentous bacteria belonging to the Bacteroidetes present in activated sludge plants. Microbiology. 2008;**154**:886–894.
33. Schütze A, Benndorf D, Püttker S, Kohrs F, Bettenbrock K. The impact of ackA, pta, and ackA-pta mutations on growth, gene expression and protein acetylation in *Escherichia coli* K-12. Front Microbiol. 2020;**11**:233.
34. Kong Y, Nielsen JL, Nielsen PH. Identity and ecophysiology of uncultured actinobacterial polyphosphate-accumulating organisms in full-scale enhanced biological phosphorus removal plants. Appl Environ Microbiol. 2005;**71**:4076–4085.
35. Marques R, Santos J, Nguyen H, Carvalho G, Noronha JP, Nielsen PH, et al. Metabolism and ecological niche of *Tetrasphaera* and *Ca.* Accumulibacter in enhanced biological phosphorus removal. Water Res. 2017;**122**:159–171.
